# Review: Kirkwood–Riseman Model in Non-Dilute Polymeric Fluids

**DOI:** 10.3390/polym15153216

**Published:** 2023-07-28

**Authors:** George David Joseph Phillies

**Affiliations:** Department of Physics, Worcester Polytechnic Institute, Worcester, MA 01609, USA; phillies@4liberty.net; Tel.: +1-508-754-1859

**Keywords:** polymer solution dynamics, polymer, solution, hydrodynamics, diffusion, viscosity, hydrodynamic scaling model, models theoretical, models molecular, polymers, hydrodynamics

## Abstract

In two prior articles, I demonstrated from extensive simulational studies by myself and others that the Rouse model of polymer dynamics is invalid in polymer melts and in dilute solution. However, the Rouse model is the foundational basis for most modern theories of polymeric fluid dynamics, such as reptation/scaling models. One therefore rationally asks whether there is a replacement. There is, namely by extending the Kirkwood–Riseman model. Here, I present a comprehensive review of one such set of extensions, namely the hydrodynamic scaling model. This model assumes that polymer dynamics in dilute and concentrated solution is dominated by solvent-mediated hydrodynamic interactions; chain crossing constraints are taken to create only secondary corrections. Many other models assume, contrariwise, that in concentrated solutions, the chain crossing constraints dominate the dynamics. An extended Kirkwood–Riseman model incorporating interchain hydrodynamic interactions is developed. It yields pseudovirial series for the concentration and molecular weight dependencies of the self-diffusion coefficient Ds and the low-shear viscosity η. To extrapolate to large concentrations, rationales based on self-similarity and on the Altenberger–Dahler positive-function renormalization group are presented. The rationales correctly predict how $D_{s}$ and η depend on polymer concentration and molecular weight. The renormalization group approach leads to a two-parameter ansatz that correctly predicts the functional forms of the frequency dependencies of the storage and loss moduli. A short description is given of each of the papers that led to the hydrodynamic scaling model. Experiments supporting the aspects of the model are noted.

## 1. Introduction

### 1.1. The Hydrodynamic Scaling Model

In two prior articles [1,2], we considered the simulational tests of the Rouse–Zimm [3,4] and Kirkwood–Riseman [5] models for chain dynamics in polymeric fluids. We demonstrated that the behavior of model polymer chains in simulated melts and simulated dilute solutions under shear was entirely inconsistent with the Rouse model. To the very limited extent that the simulations asked the necessary questions, polymer behavior was found to be consistent with the Kirkwood–Riseman model. It was, however, appropriate to ask whether there are good tests of the Kirkwood–Riseman model, and whether the model passes those tests. This review article treats that question, finding that appropriate tests [6] do indicate the validity of the Kirkwood–Riseman model as extended to non-dilute solutions.

The original model of Kirkwood and Riseman referred to a single-polymer molecular moving through a Newtonian fluid. Historically, it was reasonable to attempt to extend the model to calculate, e.g., the concentration dependence of the viscosity η(c). Note related papers by Brinkman [7], Riseman and Ullmann [8], Saito [9,10], Yamakawa [11], Freed and Edwards [12,13,14], Freed and Perico [15], and Altenberger et al. [16]. There was awareness in these reports that the long-range nature of hydrodynamic interactions can lead to improper integrals in generating pseudovirial series for η(c). Edwards and Freed proposed [12,13,14] that the needed integrals were in fact proper due to a hypothesized process of hydrodynamic screening, but later calculations by Freed and Perico [15] and by Altenberger et al. [16] demonstrated that there is in fact no such phenomenon as hydrodynamic screening in polymer solutions.

In the decades since the aforementioned work was performed, scientific interest shifted from hydrodynamic models to the tube/reptation models of polymer dynamics. In many of these models, polymer motion over short time periods, and polymer motion (’reptation’) through the hypothesized tubes in entangled polymeric fluids, were assumed to be described by Rouseian dynamics. However, as we previously found [1,2], simulations show that the Rouse model does not describe polymer motions in the melt.

At one time, it appeared plausible that the tube model for polymer melts could also be applied to polymer solutions [17]. Reviews [18,19,20] instead concluded that reptation/tube/scaling models are not applicable to polymer solutions, at least for the solutions of polymers in commonly studied concentration and molecular weight ranges. A monograph-length examination of a wide range of polymer solution properties [21] came to a similar conclusion.

This review considers an approach that effectively extends the Kirkwood–Riseman model from a dilute solution to concentrated solutions. The results are collectively described as the hydrodynamic scaling model. This model provides an alternative to tube/reptation models, which assume the use of Rouseian dynamics. The Kirkwood–Riseman and tube/reptation models differ in their assumptions as to the important forces between polymers and as to their domains of validity. There are two intermolecular forces under consideration, namely topological forces (chain crossing constraints) and solvent-mediated hydrodynamic forces. Reptation models take chain crossing constraints to be the dominant interaction and hydrodynamic interactions to provide at most secondary corrections. The hydrodynamic scaling model takes hydrodynamic forces to be the dominant interaction and chain crossing constraints to provide secondary corrections. Tube/reptation models refer to entangled polymer systems, systems in which the polymer concentration and molecular weight are large enough that chain motion is confined to tubes formed by neighboring chains. Tube/reptation models are not applicable to unentangled polymer systems, in which the polymers are too short or too dilute to form tubes. The hydrodynamic scaling model is applicable to dilute as well as non-dilute solutions of polymers having any molecular weight, small or large.

The hydrodynamic scaling model for the dynamics of non-dilute polymer solution has been presented in an extended series of papers [6,20,21,22,23,24,25,26,27,28,29,30,31,32,33,34,35,36,37,38,39,40,41,42,43,44,45,46,47]. The objective here is to present the results of these papers in a coherent way, showing what has been calculated thus far and what remains to be accomplished. The model arises from the Kirkwood–Riseman model [5]; it transcends the earlier work of Kirkwood and Riseman by including hydrodynamic interactions between different polymer molecules.

Five major components of the model are readily identified:

**First**, the hydrodynamic scaling model presumes that the dominant interactions between neutral polymers in solution are the solvent-mediated hydrodynamic forces. Chain crossing constraints are taken to provide at most secondary corrections. How is this possible? Because hydrodynamic forces are strong, the nearby segments of different polymer molecules move in unison with each other, so the effects of chain crossing constraints are greatly reduced. When two chains are close to each other, each chain drags the other along, rather than each chain acting as a stationary obstacle to block the other chain’s movements.

**Second**, following the Kirkwood–Riseman [5] model, each polymer chain is treated as a line of frictional centers (“beads”) separated by a series of frictionless links (“springs”). The hydrodynamic interactions between beads on different chains are taken to be described by the Oseen tensor [4] and its modern short-range extensions [48].

**Third**, the above assumptions are used to obtain a pseudovirial expansion for the concentration dependence of each transport coefficient, as a power series in concentration.

**Fourth**, to extend the model to elevated concentrations, we have recourse to self-similarity [23] or to renormalization group methods [41]. The renormalization group method of choice is the Altenberger–Dahler positive function renormalization group [49,50,51,52,53]. Altenberger and Dahler developed this group from Shirkov’s general treatment of renormalization analysis, based on functional self-similarity [54,55,56]. While renormalization group methods are indirect, they allow one to extrapolate lower-order pseudovirial expansions to elevated concentrations.

**Fifth**, the quantitative success of the hydrodynamic scaling model is in part based on polymer statics. In particular, it has been theoretically predicted [57] and experimentally demonstrated [57,58] that, in solution, polymer coils contract as the polymer concentration is increased. This fairly modest degree of chain contraction has a substantial effect on the predicted concentration dependencies of the polymer transport coefficients.

The hydrodynamic scaling model was first used to treat the self-diffusion coefficient $D_{s}$ of polymers in solution, predicting the functional form for the dependence of $D_{s}$ on polymer concentration *c* and polymer molecular weight *M*. Physical interpretations and predictions of numerical values for the functional form’s parameters have been provided [23,27,28,29]. The model has been extended to consider the effect of polymer concentration on the mobility of the individual beads of a polymer chain and on the mobility of small probe molecules in the surrounding solution [35]. An extended calculation predicted the low-shear viscosity of non-dilute polymer solutions [45]. The consideration of the inferred fixed-point-structure of the renormalization group led to an ansatz [42] that qualitatively determines the frequency dependencies of the storage and loss moduli. The validity of the hydrodynamic scaling model is shown by a huge mass of experimental data, as found in my companion volumes *Phenomenology of Polymer Solution Dynamics* [21,59]. In the following, the discussion of experiments will be limited to results that test particular aspects of the hydrodynamic scaling model.

### 1.2. Reptation/Scaling and Hydrodynamic Scaling Models Compared

This section compares the reptation/scaling and hydrodynamic scaling models. The major emphasis is on points where the two models are entirely different. Failure to recognize the great disparities between the two models occasionally leads to confusion in the literature. Readers should recognize that there are large numbers of modestly different reptation/scaling treatments and several different hydrodynamic treatments.

The core physical difference between the reptation/scaling and hydrodynamic scaling treatments is that the models do not agree as to which forces dominate the polymer solution dynamics. Many models [17] assume that, at elevated concentrations, chain crossing (topological) constraints (“entanglements”) between polymer chains are the dominant physical interactions. In these models, hydrodynamic interactions between chains serve primarily to dress the bare monomer drag coefficients. Hydrodynamic scaling models assert, to the contrary, that hydrodynamic forces are dominant. In these models, excluded-volume and chain crossing constraints are taken to provide only secondary corrections to the hydrodynamic interactions. The hydrodynamic scaling model is not unique in assuming the dominance of hydrodynamic interactions. Oono’s renormalization group treatment of mutual diffusion shares with the hydrodynamic scaling model the assumption that hydrodynamic forces are dominant [60].

Corresponding to the assumptions as to the nature of the dominant forces, there are assumptions as to the concentration ranges in which the models are valid. Reptation/scaling models require that the concentration is large enough that neighboring polymer coils overlap with each other and form *entanglements*, circumstances where chain crossing constraints are particularly significant. As a result, there is a lowest concentration $c^{*}$, the overlap concentration, below which tube model/reptation models are inappropriate. Tube models describe small concentrations $c<c^{*}$ as constituting the *dilute* regime, while in the reptation models, concentrations $c>c^{*}$ include the overlapping *semidilute*, *entangled*, and *concentrated* regimes.

Entanglements are not significant in the hydrodynamic scaling model, whose validity extends up from extreme dilution toward the melt. However, within the hydrodynamic scaling model, a transition concentration regime is expected, above which typical gaps between polymer chains are similar in size to individual solvent molecules. At larger concentrations, the typical gaps are smaller than solvent molecules, so that it appears inappropriate to describe solvent dynamics in terms of continuum fluid mechanics.

Entanglement-based models were originally applied to described the diffusion of a single polymer molecule, the *probe* chain, through a chemically cross-linked gel, the polymer *matrix*. In a cross-linked gel, the chains of the matrix cannot move over large distances [61]. Probe chains must thread their way through the matrix, like a very long snake threading its way through a grove of bamboo.

To transfer the entanglement model from probe chains in a cross-linked gel to probe chains in a polymer solution, it was hypothesized that the motions of probe chains in cross-linked gels and in polymer solutions can be given the same description. Unlike a gel, in the solution, the matrix chains are free to move. Entanglement-based models assume that, on the time scales of interest, these being the time scales on which the probe chains move, the matrix chains are effectively stationary. The entangled matrix chains of a polymer solution are said to form a *transient lattice* or *pseudogel* that constrains probe chain motions in the same way that a true cross-linked gel constrains probe chain motions, namely the probe chain can only move parallel to its own chain contour.

In the hydrodynamic scaling model, there is no transient lattice or pseudogel. In non-dilute solutions and melts, chains remain free to translate and to rotate around their center of masses, though their motions are delayed by other neighboring chains.

In the tube/reptation models, it is implicitly assumed that, when the probe chain encounters some neighboring matrix chains, the probe chain does not drag the matrix chain along; instead, the probe chain is brought to a stop by the matrix chain. No rationale for this implicit assumption is provided. It is thus assumed in reptation-scaling models that a long polymer chain in a non-dilute polymer solution can only move through solution in the ways that the chain can move through a true cross-linked gel, namely over large distances, and the probe chain only moves parallel to its own length. The hydrodynamic scaling models make an opposite assumption, namely that when polymer chains encounter each other, the chain segments on neighboring chains tend to move in parallel directions, so they do not block each others’ motions.

Many entanglement-based models incorporate a second, independent assumption, the *scaling* assumption, which proposes that polymer transport coefficients such as the self-diffusion coefficient $D_{s}$ depend on polymer concentration *c* and polymer molecular weight *M* via scaling laws, e.g.,

(1) $$D_{s}\left(c,M\right)=D_{cm}c^{\nu }M^{\gamma },$$

where ν and γ are scaling exponents. The business of entanglement models and experimental studies is then to calculate or measure the exponents ν and γ. Presumably, a complete model would also compute the scaling prefactor $D_{cm}$ and supply the ranges of *c* and *M* for which the model should be accurate, but much early work treated $D_{cm}$ as an undetermined constant.

The hydrodynamic scaling model usually predicts stretched exponentials:

(2) $$D_{s}\left(c,M\right)=D_{o}\exp\left(-\alpha c^{\nu }M^{\gamma }\right).$$

This functional form theoretically arises from the Altenberger–Dahler positive-function renormalization group, when it is used to extrapolate $D_{s}\left(c,M\right)$ from smaller to larger concentrations, as treated in Section 6. The model quantitatively predicts ν and γ, quantitatively predicts the molecular weight dependence of α, and reduces the calculation of α to a single parameter *a*, the same *a* describing η(c,M) and $D_{s}\left(c,M\right)$.

### 1.3. Historical Matters Aside

The hydrodynamic scaling model arose from a series of entirely empirical observations. Experimental studies of the diffusion of microscopic polystyrene latex spheres (as probes) through solutions of non-neutralized polyacrylic acid, poly-ethylene oxide, and bovine serum albumin (as matrices) [62,63,64,65,66,67] found that the concentration dependence of the probe’s diffusion coefficient $D_{p}$ could be described to good accuracy by stretched exponentials in polymer concentration, *viz.*,

(3) $$D_{p}\left(c\right)=D_{o}\exp\left(-\alpha c^{\nu }\right).$$

where *c* is the polymer concentration, $D_{o}$ is the probe diffusion coefficient in the limit of low concentration, and in the original work, α and ν were fitting parameters. The comparison of these experimental results [22] revealed that ν was consistently in the range of 0.5–1.0, while over two orders of magnitude in the polymer molecular weight *M*, one had:

(4) $$\alpha ∼M^{\gamma },$$

for γ=0.9±0.1. Measurements with different probe sizes found that α is approximately independent of the probe sphere radius *R*.

Furthermore, in most of these systems, $D_{p}$ did not track the solution viscosity via $D_{p}∼\eta^{-1}$. In this non-Stokes–Einsteinian behavior, probes diffused faster than expected from their known sizes and the solution viscosity. Obvious artifacts, including polymer adsorption by the spheres and polymer-driven sphere aggregation, would cause the spheres to diffuse slower than expected, indicating that this non-Stokes–Einsteinian behavior was not simply an artifact. Non-Stokes–Einsteinian behavior, which was noticed well before Equation (3) and the dependencies of α and ν on *M* and *R* were identified, was the driving motivation for the previous [62,63,64,65,66,67] experimental work.

Equation (3) was then compared [20] with the published studies of the polymer self-diffusion coefficient $D_{s}$, finding that $D_{s}\left(c\right)$ uniformly follows a similar equation:

(5) $$D_{s}\left(c\right)=D_{o}\exp\left(-\alpha c^{\nu }\right).$$

Equation (5) was therefore identified [20] as the *universal scaling equation* for polymer self-diffusion. The functional form of Equation (3) has since been tested [25,37,39] against the literature reports of the polymer solution viscosity η, sedimentation coefficient *s*, rotational diffusion coefficient $D_{r}$, and the dielectric relaxation time $\tau_{r}$. In each case, these transport coefficients have stretched exponential concentration dependencies with various prefactors and exponents α and ν.

Several features of Equation (3), as revealed in Refs. [20,22], were not in accordance with expectations from entanglement-based models of polymer solution dynamics. In particular: (i) The concentration dependence was found to be a stretched exponential in *c*, not the expected power law in *c*; (ii) The concentration dependence was described over all concentrations studied by a single set of parameters (α,ν), with no indication of a transition in dynamic behavior between a “dilute” regime (in which hydrodynamics was expected to dominate) and a “semidilute” regime (in which polymer coils overlapped and entanglements were proposed to dominate); (iii) For probe diffusion (spheres diffusing through random-coil polymers), in the semidilute regime $D_{p}\left(c\right)$ was found to be dependent on—rather than independent of—polymer molecular weight; (iv) In the semidilute regime, α was found to be nearly independent of the probe radius, even though it had been expected to have a strong dependence on the probe radius; and (v) $D_{p}$ of large probes was expected to be determined by the macroscopic solution viscosity, which it was not. Furthermore, (vi) in the dilute solutions, $D_{s}\left(c\right)$ was often proposed in the context of reptation/scaling models to be nearly independent of *c*. None of the expectations (i)–(vi) were met in the studied systems.

How might this set of discrepancies between the universal scaling Equation (3) and expectations based on entanglement models be resolved? First, one could always propose that the agreement between the universal scaling equation and the particular datasets with which it had been compared was a curiosity, an empirical coincidence having no real importance. In that case, the equation would be an accident having no relationship to fundamental theoretical considerations. Second, one could propose that the agreement arose because the universal scaling equation is remarkably flexible. This second proposal encounters the information-theoretic obstacle that the equation has three free parameters (and the measurable zero-concentration limiting constant $D_{o}$), so it can therefore cover neither more nor less of the possible solution space than can any other reasonable three-parameter equation.

Finally, the criticism was advanced that Equation (3) is purely empirical and has no physical content. This final criticism led to the clear recommendation [68] that the proponents of Equation (3) needed to find an ab initio theoretical derivation of Equation (3), preferably a derivation that reveals the physical interpretations of α and ν. The remainder of this article reviews the research program that generated the requested derivation. Equation (3), including ab initio numerical values for α and ν and their molecular weight dependencies, was obtained. We review the papers that supplied that derivation, ending antiquated suggestions that the universal scaling equation and its parameters are purely empirical and have no physical interpretation.

### 1.4. Precis of the Work

This section presents an outline of the remainder of this article.

In Section 2, we first discuss the less-studied Kirkwood–Riseman model, because the Kirkwood–Riseman model provides the foundation for determining hydrodynamic interactions between polymer chains. As an example, the drag coefficient of a Kirkwood–Riseman polymer is calculated.

Section 3 presents our extended Kirkwood–Riseman model. The extension calculates chain–chain hydrodynamic interactions. It thus provides the physical basis for the hydrodynamic scaling model. Section 3.1 presents the modern bead–bead hydrodynamic interaction tensors including short-range and three-bead interactions. Section 3.2 shows how to move from bead–bead to chain–chain hydrodynamic interactions in the context of the Kirkwood–Riseman model.

Section 4 uses the extended Kirkwood–Riseman model to calculate, through $O(c^{2})$, the concentration dependence of the polymer self-diffusion coefficient.

Section 5 uses the model to calculate the concentration dependence of the viscosity. Section 5.1 calculates the flow field $u^{\left(1\right)}$ created by the scattering of a shear field $u^{\left(0\right)}$ by a polymer chain, and the additional flow field $u^{\left(2\right)}$ created by the scattering of flow field $u^{\left(1\right)}$ by a second polymer. Section 5.2 calculates the power dissipated by various polymer chains exposed to flow fields $u^{\left(0\right)}$, $u^{\left(1\right)}$, and $u^{\left(2\right)}$. Section 5.3 calculates the total shear field that would be experimentally determined as a result of these flow fields, leading to a determination in Section 5.4 of the intrinsic viscosity and the Huggins coefficient for the extended Kirkwood–Riseman model.

Section 6 considers paths for extending the hydrodynamic calculation of pseudovirial coefficients, as seen in Section 4 and Section 5, to determine polymer dynamics at elevated concentrations. Section 6.1 considers self-similarity rationales. Section 6.2 develops the mathematical basis for the alternative approach, the Altenberger–Dahler positive-function renormalization group.

Section 7 then uses the positive-function renormalization group to extend the calculations of Section 4 and Section 5 to large concentrations. The universal scaling equation for polymer self-diffusion is obtained.

Section 8 presents an ansatz for computing the frequency dependencies of the bulk and shear moduli. The ansatz, *two-parameter temporal scaling*, arises from the inferred fixed-point structure of the positive-function renormalization group calculation of the shear viscosity.

Section 9 offers single-paragraph summaries, in publication order, of the theoretical and phenomenological papers that describe the hydrodynamic scaling model.

Section 10 summarizes the experimental results testing various aspects of the hydrodynamic scaling model. The tests confirm the validity of the model.

Section 11 discusses the results here, and considers consider where the hydrodynamic scaling model has gaps and omissions, thereby identifying a few directions for future research.

## 2. Single-Chain Behavior

### 2.1. The Models

This section discusses models for single-chain polymer motion. There are two major classes of models, namely models based on the Kirkwood–Riseman [5] treatment, and models based on the treatments of Rouse [3] and Zimm [4]. Qualitatively, the two classes of model supply radically different descriptions for chain motion in dilute solutions. The hydrodynamic scaling model is based on extensions of the Kirkwood–Riseman model, while in contrast, many tube/reptation models reference the original Rouse treatment. I have previously discussed [1,2] the Rouse model in detail and will not repeat that discussion here. A major emphasis of this Section is therefore to alert readers familiar with Rouse and Zimm models as to the very different way in which Kirkwood and Riseman described the movements of an individual polymer coil.

In all of these models, a polymer chain is treated as a series of *beads*, pairs of beads that are connected by *links*. The polymer interacts hydrodynamically with the solvent via the beads, each of which acts as a small sphere or point that applies a frictional force on the solvent. The links are hydrodynamically inert. They serve to control the distances between the beads. In the Rouse and Zimm models, the beads are abstractions representing the hydrodynamic friction of a subsection of the polymer, while the links are treated as the subsections of the polymer chain, each subsection being barely long enough to have a Gaussian distribution of lengths. In the original Kirkwood–Riseman model, the beads were taken to be monomer units, while the links were the covalent bonds connecting one monomer to the next. In some modern applications of the Kirkwood–Riseman model, the beads and links are interpreted in the Rouse and Zimm sense.

In the Rouse and Zimm models, each subsection acts as a Hookian spring. Each subsection generates an attractive force on the two beads to which it is attached. The force has magnitude kℓ, where *k* is an effective spring constant and *ℓ* is the distance between the two beads; the force acts along the line of centers connecting the beads. In these models, the unstretched (rest) length of each subsection is zero.

In the original Kirkwood–Riseman model, the links are covalent bonds having rigid lengths and bond angles, but perhaps a potential energy for torsion. Within the model, the effect of the links is to determine the statistico-mechanical distribution functions for the distances between pairs of beads along the polymer chain. Because the beads of the original Kirkwood–Riseman model are monomers, the number of beads in a Kirkwood–Riseman chain can be very large, much larger than the number of beads in a Rouse or Zimm model for the same polymer. For beads that are well separated along the chain, in the Kirkwood–Riseman model, the distribution function for the bead–bead distance is assumed to be a Gaussian.

These models for polymer dynamics make contradictory assumptions as to which polymer chain motions are of interest in solutions. In the Kirkwood–Riseman model, the interesting motions of the beads are described as *whole-body motion*. In whole-body motion, the polymer beads may experience equal linear displacements, and they may rotate around the polymer center of mass, but the displacements and rotations are such that the chain motion does not alter the relative positions of the polymer beads. The phrase *whole-body motion* does not mean that the polymer coil is mechanically rigid. A full description of the motions of *N* polymer beads requires 3N coordinates. The whole body motion description extracts from these 3N coordinates a set of six collective coordinates, describing whole-body translations and rotations, with the remaining motions are described as the *internal modes*.

Kirkwood and Riseman are entirely specific that the polymer coil in their model has internal motions, so that the relative positions of beads fluctuate with respect to each other. However, in the Kirkwood–Riseman model, the whole-body motions are assumed to dominate polymer solution dynamics. Internal motions are taken to provide corrections to the dominant chain motions, the whole body displacements and rotations. The internal motions are coarse-grained out, so bead velocities are approximated as being the components created by the polymer translational and angular velocities. Kirkwood and Riseman did not compute the magnitude of the internal mode corrections.

In contrast to the Kirkwood–Riseman model, the Rouse and Zimm models assume that the beads move relative to each other. The relative motions of the beads are driven by attractive forces between adjoining beads, as created by the links. These relative motions are described by the Rouse–Zimm polymer internal modes, and are taken to dominate polymer solution dynamics. Rouse and Zimm model polymer coils do perform whole-body translation, but translation does not to contribute to the polymer solution’s viscosity.

### 2.2. Kirkwood–Riseman Model

We consider the Kirkwood–Riseman model [5], whose ansatz provides the basis of the hydrodynamic scaling model. The Kirkwood–Riseman model is much less discussed than the Rouse and Zimm models and their extensions are, in part because it is more mathematically demanding, and in part because Kirkwood and Riseman used a less familiar notation. This presentation of the Kirkwood–Riseman model has therefore been reset in a more modern form.

The Kirkwood–Riseman model describes a chain of *N* beads connected by links having a length of $b_{0}$. The links are covalent bonds, with adjoining links separated by a rigid angle θ. Successive three-bead planes are related by a torsion angle ϕ. In the original model, the potential energy was taken to be independent of the angle ϕ. The effective bond length, the contribution of each link to the distance between distant beads, is:

(6) $$b=\left(\frac{1+\langle \cos(\phi )\rangle }{1-\langle \cos(\phi )\rangle }\right)\left(\frac{1-\cos(\theta )}{1+\cos(\theta )}\right)b_{0}.$$

For beads *ℓ* and *s* that are well separated, Kirkwood and Riseman supply several average values, notably:

(7) $$\begin{array}{cc} \langle |R_{\ell s}{|}^{2}\rangle & =|\ell -s|b^{2}, \end{array}$$

(8) $$\begin{array}{cc} \langle |R_{0\ell }{|}^{2}\rangle & =b^{2}\left(\frac{12\ell^{2}+N^{2}-2N+1}{12(N-1)}\right), \end{array}$$

(9) $$\begin{array}{cc} \langle R_{0\ell }\cdot R_{0s}\rangle & =\frac{b^{2}}{N-1}\left(\frac{\ell^{2}+s^{2}}{2}-\frac{N-1}{2}|\ell -s|+\frac{{\left(N-1\right)}^{2}}{2}\right), \end{array}$$

(10) $$\begin{array}{cc} \langle \frac{1}{R_{\ell s}}\rangle & =\frac{6}{\sqrt{\pi }b|\ell -s{|}^{1/2}}. \end{array}$$

Here, beads *ℓ* and *s* have locations $r_{\ell }$ and $r_{s}$, $R_{\ell s}=r_{s}-r_{\ell }$ is the vector from bead *ℓ* to bead *s*, $R_{\ell s}=\left|R_{\ell s}\right|$, and $r_{0}$ is the location of the center of mass of the polymer, so that $R_{0\ell }$ is the vector from the center of mass to bead *ℓ*. The final equation assumes that $R_{\ell s}$ has a normal distribution.

The Kirkwood–Riseman model assumes that polymer beads have a long-range hydrodynamic interaction, as described by the Oseen tensor:

(11) $$T_{ij}\left(r_{ij}\right)=\frac{1}{8\pi \eta_{0}r_{ij}}\left(I+{\hat{r}}_{ij}{\hat{r}}_{ij}\right),$$

which gives the fluid flow created at a point $r_{j}$ by a force $F_{i}$ applied to the solution at point $r_{i}$. The vector from point *i* to point *j* is $r_{ij}$, with magnitude $r_{ij}=\left|r_{ij}\right|$ and corresponding unit vector ${\hat{r}}_{ij}=r_{ij}/r_{ij}$. Here, $\eta_{0}$ is the solvent viscosity. In Equation (11) and its associated notation, there is no assumption that there is a polymer bead at point $r_{j}$. The theoretical model treats the force as a point source, and assumes that the presence of the polymer has no effect on the solvent’s viscosity, an assumption that is experimentally known to be incorrect [69,70,71,72]. The fluid flow induced at $r_{j}$ by $F_{i}$ is:

(12) $$v^{\prime }\left(r_{j}\right)=T_{ij}\left(r_{ij}\right)\cdot F_{i}\left(r_{i}\right).$$

Within the model, the forces $F_{i}$ arise because the beads are moving with respect to the fluid. If a bead is stationary with respect to the local fluid flow, it exerts no force on the fluid. The force exerted on the fluid by a bead *ℓ* is determined by the velocity $u_{\ell }$ of the bead, the velocity $v(r_{\ell })$ that the fluid would have had, at the point $r_{\ell }$, if the bead were not present, and the drag coefficient ξ of the bead, namely:

(13) $$F_{i}=\xi \left(u_{\ell }-v\left(r_{\ell }\right)\right).$$

Because the beads are treated as points, a single bead is assumed to exert no torque on the surrounding fluid.

We now come to the modeled dynamics of the polymer. The beads are taken to lie along a Gaussian chain, meaning that, on average, their concentration declines with the distance from the center of mass, as a Gaussian in that distance. The velocities of the individual beads are taken to be entirely determined by the time-dependent chain center-of-mass velocity V(t) and chain rotational velocity Ω(t) as:

(14) $$u_{\ell }\left(t\right)=V\left(t\right)+\Omega \left(t\right)\times R_{0\ell }.$$

$u_{\ell }$, as given by Equation (13), is the velocity that the bead *ℓ* would have, if it were part of a rigid body that had translational velocity V and rotational velocity Ω. We therefore describe the chain motions as whole-body translation and whole-body rotation. As noted above, Kirkwood and Riseman recognized that polymer molecules also have internal coordinates whose fluctuations contribute to the bead velocities, leading to an extra velocity component, different for each bead, in Equation (14), but those components were neglected as an approximation.

What forces act on a polymer chain? The model assumption is that, in the absence of external forces, over long times, the polymer’s translational and rotational accelerations must both average to zero. Under these conditions, the long-time averages of the sum of the forces and of the sum of the torques on each chain must both vanish. The zero-force and zero-torque conditions determine the response of the polymer to an external force or to an external torque.

As an example of the effect of hydrodynamic interactions, we consider the drag coefficient (and hence the diffusion coefficient) of a polymer chain. The analysis of Zwanzig [73] is followed. Note that Kirkwood and Riseman took $F_{j}$ to be the force on the solvent, while Zwanzig takes $F_{j}$ to be the force on the bead, so the papers have sign differences. We have a polymer chain whose beads have arbitrary velocities $u_{\ell }$, while the fluid at $r_{\ell }$ has an unperturbed velocity $v_{\ell }^{0}$. The hydrodynamic interactions perturb the fluid flow at $r_{\ell }$, so the actual fluid velocity at $r_{\ell }$ is:

(15) $$v_{\ell }=v_{\ell }^{0}+\sum_{k\neq \ell =1}^{N}T_{\ell k}\cdot F_{k}.$$

However, the hydrodynamic force that a bead *k* exerts on the solvent is:

(16) $$F_{k}=f\left(u_{k}-v_{k}\right),$$

where *f* is the drag coefficient of a single bead. Combining the above two equations,

(17) $$v_{\ell }=v_{\ell }^{0}-\sum_{k\neq \ell =1}^{N}T_{\ell k}\cdot f\left(v_{k}-u_{k}\right).$$

Subtracting $u_{\ell }$ from each side of the equation,

(18) $$v_{\ell }-u_{\ell }=v_{\ell }^{0}-u_{\ell }-\sum_{k\neq \ell =1}^{N}T_{\ell k}\cdot f\left(v_{k}-u_{k}\right).$$

which allows us to write:

(19) $$v_{\ell }^{0}-u_{\ell }=f\sum_{k=1}^{N}\mu_{\ell k}\cdot \left(v_{k}-u_{k}\right).$$

The new matrix μ is:

(20) $$\mu_{\ell k}=\frac{I\delta_{\ell k}}{f}+T_{\ell k},$$

where the rule $T_{kk}=0$ has been applied and I is the 3×3 identity matrix.

Matrix inversion gives the $v_{k}-u_{k}$ in terms of the $v_{\ell }^{0}-u_{\ell }$ and the inverse of μ, namely:

(21) $$v_{k}-u_{k}=f^{-1}\sum_{\ell =1}^{N}{\left(\mu^{-1}\right)}_{k\ell }\cdot \left(v_{\ell }^{0}-u_{\ell }\right),$$

so the force on a bead *k* due to its hydrodynamic interactions with the solvent becomes:

(22) $$-F_{k}\equiv f\left(v_{k}-u_{k}\right)=-\sum_{\ell =1}^{N}{\left(\mu^{-1}\right)}_{k\ell }\cdot \left(v_{\ell }^{0}-u_{\ell }\right).$$

The minus sign appears because $F_{k}$ is the force of the bead on the solvent, not vice versa.

The drag coefficient $f_{c}$ of the polymer chain is obtained by choosing all bead velocities to be equal to $u_{0}$ and the unperturbed fluid velocity to be zero, and calculating the total of the drag forces on all beads of the chain, leading to:

(23) $$-\sum_{k=1}^{N}F_{k}\equiv f_{c}u_{0}=\sum_{k=1}^{N}\sum_{\ell =1}^{N}{\left(\mu^{-1}\right)}_{k\ell }\cdot u_{0}.$$

Bead–bead hydrodynamic interactions, as described by the Oseen tensor, thus perturb the drag coefficient of the whole chain.

## 3. Extended Kirkwood–Riseman Model

Here, we consider the extension of the Kirkwood–Riseman model to treat multiple polymer chains. The calculation refers to time scales that are sufficiently long that polymer inertia can be neglected. The solvent is treated as a continuum fluid. Each polymer chain is treated as a line of beads that interacts with the solvent by applying to the solvent a series of point forces. The point forces create solvent flows and hydrodynamic forces on other polymer beads, the flows and forces being described by mobility tensors $\mu_{ij}$. Beads on each chain are linked by springs; a spring is a hydrodynamically inert coupler that determines the distribution of bead–bead distances. We only consider *ghost chains* that can pass through each other; excluded volume interactions only serve to set the minimum distances of approach between pairs of beads. Chain motions are approximated by whole-chain translation and rotation; internal modes that change the shape of a chain have not yet been included in the hydrodynamic scaling model.

### 3.1. Bead–Bead Hydrodynamic Interactions

The effects of hydrodynamic interactions are usefully described by mobility tensors $\mu_{ij}$. These tensors give the hydrodynamic force on a bead (or chain) *i* due to the force a bead (or chain) *j* exerts on the solvent; i=j is allowed. Separate expressions are needed for the self (i=j) and distinct (i≠j) components of $\mu_{ij}$. For the calculations here, we begin with the $\mu_{ij}$ that relate the force on bead *i* to the force that bead *j* applies to the solvent. After some work, we end with a second set of mobility tensors that give the force and torque on a chain *i* due to a force or torque applied to the solution by a chain *j*.

The mobility tensors are specifically of interest because they determine the self-diffusion coefficient via:

(24) $$D_{s}=\frac{1}{3}k_{B}T\;\mathrm{trace}\left(\mu_{ii}\right).$$

Here, $k_{B}$ is Boltzmann’s constant and *T* is the absolute temperature. For spheres in solution, the mobility tensors can be expanded as power series in a/r, where *a* is a sphere radius and *r* is the distance between the spheres, as developed by Kynch [48], Mazur and van Saarloos [74], this author [75], and Ladd [76]. Part of the expansion improves the accuracy of the hydrodynamic interaction tensor for spheres that are close to each other. Other extensions describe additional interactions between three or more spheres. The lowest-order approximation to the hydrodynamic interaction between two spheres is the Oseen tensor. The $\mu_{ij}$ can be expanded as [48,74,75]:

(25) $$\mu_{ii}=\frac{1}{f_{o}}\left(I+\sum_{l,l\neq i}b_{il}+\sum_{\begin{array}{c} m,m\neq i\;\mathrm{or}\;l\\ l\neq i \end{array}}b_{iml}+...\right)$$

for the self terms, and:

(26) $$\mu_{ij}=\frac{1}{f_{o}}\left(T_{ij}+\sum_{\begin{array}{c} i,j,m\\ i,j,m\;\mathrm{distinct}\; \end{array}}T_{imj}+...\right),\;i\neq j$$

for the distinct terms.

The leading terms of the b and T tensors are [74]:

(27) $$b_{il}=-\frac{15}{4}{\left(\frac{a}{r_{il}}\right)}^{4}{\hat{r}}_{il}{\hat{r}}_{il},$$

$$b_{iml}=\frac{75a^{7}}{16r_{im}^{2}r_{il}^{2}r_{ml}^{3}}\{\left[1-3{\left({\hat{r}}_{im}\cdot {\hat{r}}_{ml}\right)}^{2}\right]\left[1-3{\left({\hat{r}}_{ml}\cdot {\hat{r}}_{li}\right)}^{2}\right]$$

(28) $$+6\left({\hat{r}}_{im}\cdot {\hat{r}}_{ml}\right){\left({\hat{r}}_{ml}\cdot {\hat{r}}_{li}\right)}^{2}-6\left({\hat{r}}_{im}\cdot {\hat{r}}_{ml}\right)\left({\hat{r}}_{ml}\cdot {\hat{r}}_{li}\right)\left({\hat{r}}_{li}\cdot {\hat{r}}_{im}\right)\}{\hat{r}}_{im}{\hat{r}}_{li},$$

(29) $$T_{ij}=\frac{3}{4}\frac{a}{r_{ij}}\left[I+{\hat{r}}_{ij}{\hat{r}}_{ij}\right],$$

(30) $$T_{iml}=-\frac{15}{8}\frac{a^{4}}{r_{im}^{2}r_{ml}^{2}}\left[I-3{\left({\hat{r}}_{im}\cdot {\hat{r}}_{ml}\right)}^{2}\right]{\hat{r}}_{im}{\hat{r}}_{ml},$$

where only the lowest order term (in $\frac{a}{r}$) of each tensor is shown. See Mazur and van Saarloos [74] for the higher-order terms. Here, I is the unit tensor, r=|r|, the unit vector is $\hat{r}=r/r$, $\eta_{o}$ is the solvent viscosity, and $\hat{r}\hat{r}$ is an outer product.

$b_{ij}$ and $T_{ij}$ describe the hydrodynamic interactions of a pair of interacting spheres. $T_{ij}/f_{o}$ describes the velocity induced in particle *i* due to a force applied to particle *j*, while $b_{ij}$ describes the retardation of a moving particle *i* due to the scattering by particle *j* of the wake set up by *i*. $T_{iml}$ and $b_{iml}$ describe the interactions between trios of interacting spheres. $T_{iml}$ describes the velocity of particle *i* by a hydrodynamic wake set up by particle *l*, the wake being scattered by an intermediate particle *m* before reaching *i*. $b_{iml}$ describes the retardation of a moving particle *i* due to the scattering, first by *m* and then by *l*, of the wake set up by *i*.

In most of the following, the individual beads are taken to be small relative to the distances between beads on different polymer chains, so only the lowest-order (in a/r) term is used to describe the bead–bead interactions, this being the Oseen tensor of Equation (29).

### 3.2. Chain–Chain Hydrodynamic Interactions

Having considered the hydrodynamic interactions between polymer beads, we now advance to calculate the hydrodynamic interactions between the pairs of polymer chains. The method of reflections is used to compute the interchain hydrodynamic interactions. A chain whose beads move with respect to the solvent creates flows in the surrounding solvent. These flows act on other chains. In response to those flows, the other chains move. Those chain motions induce additional solvent flows. The hydrodynamic equations are linear, so if a chain *A* is subject to flows due to chains *B* and *C*, the flow acting on chain *A* is the sum of the flows created by *B* acting on *A* and by *C* acting on *A*. Because the flow properties are linear, all hydrodynamic effects can be obtained by considering a line of chains, with each chain acting on the next in line. We say that the process is *scattering*: The flow created by each chain is *scattered* when it encounters the next chain in the line. It is not assumed that each chain in a line must be different from all the other chains in a line; the line of chains may loop back on itself so that a given chain appears in the line more than once. A crude image of the hydrodynamic effect of one moving chain on the next is given by this paper’s graphical abstract. A chain (left, blue line) moves (green vertical arrow) and puts forces on the fluid. In response, the fluid moves, symbolically represented by the horizontal arrow labeled “T”. The second chain (right, red line) responds by translating and rotating (two green arrows).

The chains in a line are labeled 1, 2, 3, …. The center-of-mass location of chain *j* is the vector $a_{j}$, with *j* labeling which of the $N_{c}$ chains is involved. The location of a bead *i* with respect to its chain’s center-of-mass is $s_{i}$. Each step of the calculation here involves only beads on a single chain, so $s_{i}$ does not need a separate label specifying the chain of which it is a part. The vectors from the center of mass of each chain in the line to the next chain’s center of mass are the vectors $R_{j}$, with $R_{j}=a_{j+1}-a_{j}$. Solvent flows are denoted $u^{\left(n\right)}\left(r\right)$; they are implicit functions of position even if no dependence on r is specified. An imposed solvent flow, such as a fluid shear field, is denoted $u^{\left(0\right)}$; in a quiescent liquid, $u^{\left(0\right)}=0$. Solvent flows created by the first, second, … chains in a sequence are denoted $u^{\left(1\right)}$, $u^{\left(2\right)}$, …, respectively.

The velocity $v_{j}$ of a bead *j* that is located on chain *i* may be divided between the center-of-mass motion, whole-body rotation, and internal mode motions as:

(31) $$v_{j}=V^{\left(i\right)}+\Omega^{\left(i\right)}\times s_{j}+{\dot{w}}_{j}.$$

Here, the chain’s center-of-mass velocity is $V^{\left(i\right)}$, the chain’s angular velocity around its center of mass is $\Omega^{\left(i\right)}$, and the bead motions arising from chain internal modes are denoted by ${\dot{w}}_{j}$. The superscripts on V and Ω identify the reflection that created those parts of V and Ω.

The chain center-of-mass velocity is:

(32) $$V^{\left(i\right)}=\frac{\partial a_{i}}{\partial t}.$$

$V^{\left(i\right)}$ is determined by averaging over the *N* beads of chain *i*, namely:

(33) $$V^{\left(i\right)}=\frac{1}{N}\sum_{j=1}^{N}v_{j}.$$

The $V^{\left(i\right)}$ and ${\dot{w}}_{i}$ are independent of $\Omega^{\left(i\right)}$, so $\Omega^{\left(i\right)}$ can be determined from Equation (31) as:

(34) $$\frac{1}{N}\sum_{j=1}^{N}s_{j}\times \left(\Omega^{\left(i\right)}\times s_{j}\right)=\frac{1}{N}\sum_{j=1}^{N}s_{j}\times v_{j}.$$

The instantaneous-square chain radius $s^{2}$ is $N^{-1}\sum_{j=1}^{N}s_{j}^{2}$.

The model describes the low-frequency regime. The chain linear and angular momenta fluctuate, but over the time scales of interest herein, the fluctuations average towards zero. For the same reason, contributions to fluid flow from the higher-frequency ${\dot{w}}_{i}$ are not taken into account. If the fluctuations in the total linear momentum and total angular momentum of each chain average towards zero, from the fundamental mechanics the total force and total torque on each chain after the first must also average towards zero. (The first chain in a line may also be subject to external forces, torques, or fluid flows, and so is a special case.) One obtains:

(35) $$\sum_{j=1}^{N}f_{j}\left(v_{j}-u\left(r_{j}\right)\right)=0,$$

and:

(36) $$\sum_{j=1}^{N}f_{j}s_{j}\times \left(v_{j}-u\left(r_{j}\right)\right)=0,$$

The four Equations (33)–(36) take us from the fluid velocity $u^{\left(n-1\right)}\left(r_{j}\right)$ at the beads of chain *n* to the center-of-mass translational and rotational velocities $V^{\left(n\right)}$ and $\Omega^{\left(n\right)}$ of chain *n*. The $V^{\left(n\right)}$ and $\Omega^{\left(n\right)}$ depend on the relative positions of the chains.

For the calculation of the self-diffusion coefficient, the first chain in the series is presumed to have some initial velocity that corresponds to its performing translational motion. For the calculation of the viscosity increment, the first chain in the series finds itself in a velocity shear. As will be seen, each chain moves at the local flow velocity. Each chain rotates so as to attempt to comply at its every point with the imposed shear flow. Each chain can translate and rotate, but its local velocity at every bead cannot be the same as the velocity that the fluid would have had at the same point if the chain were absent.

## 4. Extended Kirkwood–Riseman Model: Self-Diffusion

We now implement the method of reflections as described above. We begin with polymer chain 1 that has linear velocity $V^{\left(1\right)}$ and angular velocity $\Omega^{\left(1\right)}$ with respect to the unperturbed and hence quiescent solvent. A bead *j* on chain 1 then has the velocity $v_{j}^{\left(1\right)}=V^{\left(1\right)}+\Omega^{\left(1\right)}\times s_{j}$, plus a component corresponding to the internal modes that we are neglecting. The flow $u^{\left(1\right)}$ induced at r by all *M* beads of chain 1 is:

(37) $$u^{\left(1\right)}\left(r\right)=\sum_{j=1}^{M}T\left(r-s_{j}\right)\cdot v_{j}^{\left(1\right)}.$$

In the spirit of the Kirkwood–Riseman calculation, we now average over detailed relative locations of the individual beads. Functions of the vector s from the center of mass replace the functions of the bead label *j*. All sums $\sum_{j}f_{j}$ over beads are replaced with the integrals ∫dsg(s)f(s), s being a vector from the chain center of mass to a point within the chain, with g(s) being the density of beads at s, and f(s) being the effective drag coefficient of the beads at s. The integral of f(s) over the complete chain is the total drag coefficient $F_{o}$. Correlations in the shapes of nearby chains are neglected.

A series expansion for the Oseen tensor is $T\left(r-s\right)=T\left(r\right)-s\cdot \nabla T\left(r\right)+O\left(s^{2}\right)$, namely:

(38) $$T\left(r-s\right)=\frac{1}{8\pi \eta }\left[\frac{I+\hat{r}\hat{r}}{r}-\hat{r}\frac{s\cdot (I-3\hat{r}\hat{r})}{r^{2}}-\frac{s\hat{r}}{r^{2}}+\frac{s\cdot \hat{r}}{r^{2}}I\right]+O\left({\left(\frac{s}{r}\right)}^{2}\right).$$

The resulting induced flow field, to the lowest order in the series expansion, is:

(39) $$u^{\left(1\right)}\left(r\right)=\int ds\;g\left(s\right)\frac{f_{o}}{8\pi \eta }\left[\frac{I+\hat{r}\hat{r}}{r}-\hat{r}\frac{s\cdot (I-3\hat{r}\hat{r})}{r^{2}}-\frac{s\hat{r}}{r^{2}}+\frac{s\cdot \hat{r}}{r^{2}}I\right]\cdot \left[V^{\left(1\right)}+\Omega^{\left(1\right)}\times s_{j}\right].$$

In the above, $\int g\left(s\right)s^{2}\;ds=R_{g}^{2}$. Terms odd in s vanish by symmetry. $f_{o}$, the chain drag coefficient, is $6\pi \eta R_{h}$. By direct calculation, $\int g\left(s\right)s\cdot \hat{r}\Omega \times s\;ds=R_{g}^{2}\Omega \times \hat{r}/3$. Here, $R_{g}$ and $R_{h}$ are the radii of gyration and the hydrodynamic radius of the chain, with additional numerical subscripts on $R_{g}$ and $R_{h}$ being used to identify which chain’s radii are under consideration.

The result of these steps is:

(40) $$u^{\left(1\right)}\left(r\right)=\frac{3}{4}\frac{R_{h1}}{r}\left[\frac{I+\hat{r}\hat{r}}{r}\right]\cdot V^{\left(1\right)}+\frac{1}{2}\frac{R_{h1}R_{g1}^{2}}{r^{2}}\left(\Omega^{\left(1\right)}\times \hat{r}\right).$$

The indicated terms are the longest-range parts of the flow field created by the motions of the first chain. By expanding T(r−s) to a higher order in s·∇, one would obtain higher-order in terms ${\left(R_{g}/r\right)}^{2}$.

The calculation now proceeds by iteration. The flow field $u^{\left(1\right)}\left(r\right)$ exerts forces on the next chain in the series. The zero-force and zero-torque conditions let us calculate the linear and angular velocities $V^{\left(2\right)}$ and $\Omega^{\left(2\right)}$ of the next chain. Under the approximation that we neglect chain internal modes, the beads of the next chain move with velocities $v_{j}^{\left(2\right)}=V^{\left(2\right)}+\Omega^{\left(2\right)}\times s_{j}$. These beads cannot simply move with the solvent. As a result, the beads of chain 2 exert forces on the solvent, thereby creating a new flow field $u^{\left(2\right)}\left(r\right)$, where r is now measured from the center of mass of chain 2.

The force on a representative bead *i* of chain 2, due to the flow field $u^{\left(1\right)}\left(r\right)$ scattered by chain 1, is:

(41) $$F_{i}^{\left(2\right)}=f_{i}\left(u^{\left(1\right)}\left(R_{1}+s_{i}\right)-V^{\left(2\right)}-\Omega^{\left(2\right)}\times s_{i}\right).$$

where $f_{i}$ is the bead’s drag coefficient. The bead is at $R_{1}+s_{i}$, a displacement by $s_{i}$ from the displacement $R_{1}$ of the center of mass of chain 2 from the center of mass of chain 1.

The zero-force and zero-torque conditions are then applied to chain 2. To do this, beads at the locations $s_{i}$ are again replaced with a bead density g(s), and the flow field $u^{\left(1\right)}\left(R_{1}+s_{i}\right)$ is given a series expansion, centered on the center-of-mass of chain 2, in the powers of s·∇. The zero-force condition starts as:

(42) $$f_{o}\int ds\;g\left(s\right)\left[u^{\left(1\right)}\left(R_{1}\right)+s\cdot \nabla u^{\left(1\right)}\left(R_{1}\right)-V^{\left(2\right)}-\Omega^{\left(2\right)}\times s_{i}\right]=0,$$

while the zero-torque condition starts as:

(43) $$f_{o}\int ds\;g\left(s\right)\left[s\times u^{\left(1\right)}\left(r\right)-s\times V^{\left(2\right)}-s\times \left(\Omega^{\left(2\right)}\times s\right)\right]=0.$$

After noting that everything except s itself is independent of s, while terms odd in s integrate to zero, and integrating on s, one finds:

(44) $$V^{\left(2\right)}=u^{\left(1\right)}\left(R_{1}\right)$$

and:

(45) $$\frac{2}{3}f_{o}\Omega^{\left(2\right)}=f_{o}\int ds\;g\left(s\right)\left[s\times \left(s\cdot \nabla_{R}\right)u^{\left(1\right)}\left(R_{1}\right)\right],$$

the subscript on the **∇** being the variable with respect to which the derivatives are taken. Taking the spherical averages, one finally reaches [41]:

(46) $$\Omega^{\left(2\right)}=-\frac{3}{4}\frac{R_{h1}}{R_{1}^{2}}\left[\hat{R_{1}}\times V^{\left(1\right)}\right]-\frac{1}{4}\frac{R_{h1}R_{g1}^{2}}{R_{1}^{3}}\Omega^{\left(1\right)}\cdot \left[I-3\hat{R_{1}}\hat{R_{1}}\right].$$

The flow field due to scattering from chain 2 is:

$$u^{\left(2\right)}\left(r\right)=-\frac{9}{16}\frac{R_{h1}R_{h2}R_{g2}^{2}}{R_{1}^{2}r^{2}}\left[1-3{\left(\hat{r}\cdot {\hat{R}}_{1}\right)}^{2}\right]\left({\hat{R}}_{1}\cdot V^{\left(1\right)}\right)\hat{r}+$$

$$\frac{3}{8}\frac{R_{h1}R_{h2}R_{g1}^{2}R_{g2}^{2}}{R_{1}^{3}r^{2}}[\hat{r}\times \Omega^{\left(1\right)}-\left(\hat{r}\times {\hat{R}}_{1}\right){\hat{R}}_{1}\cdot \Omega^{\left(1\right)}+\hat{r}\cdot {\hat{R}}_{1}\left(\Omega^{\left(1\right)}\times {\hat{R}}_{1}\right)-$$

(47) $$\hat{r}\cdot \left(\Omega^{\left(1\right)}\times {\hat{R}}_{1}\right){\hat{R}}_{1}\cdot \left(I-3\hat{r}\hat{r}\right)].$$

The calculation of higher-order scattering events proceeds by iteration. From the linear and angular velocities $V^{\left(n\right)}$ and $\Omega^{\left(n\right)}$ of chain *n* in the sequence, we compute the induced fluid flow field $u^{\left(n\right)}\left(R_{n}\right)$ at the location of chain n+1. From the flow field, we compute the linear and angular velocities $V^{\left(n+1\right)}$ and $\Omega^{\left(n+1\right)}$ of chain n+1. We can now repeat the process *ad infinitum*. The final calculation only needs the part of $u^{\left(3\right)}$ created by the linear velocity $V^{\left(1\right)}$ of the first bead, namely:

$$u^{\left(3\right)}\left(r\right)=\frac{27}{64}\frac{R_{h1}R_{h2}R_{h3}R_{g2}^{2}R_{g3}^{2}}{R_{1}^{2}R_{2}^{3}r^{2}}[\left(1-3{\left({\hat{R}}_{1}\cdot {\hat{R}}_{2}\right)}^{2}\right)\times$$

(48) $$\left(1-3{\left({\hat{R}}_{2}\cdot \hat{r}\right)}^{2}-6\left({\hat{R}}_{1}\cdot {\hat{R}}_{2}\right)\left({\hat{R}}_{2}\cdot \hat{r}\right)+\hat{r}\cdot \left[I-{\hat{R}}_{2}{\hat{R}}_{2}\right]\cdot {\hat{R}}_{1}\right)]\left({\hat{R}}_{1}\cdot V_{1}\right)\hat{r}.$$

This form does not include the contribution to $u^{\left(3\right)}\left(r\right)$ from $\Omega^{\left(1\right)}$.

The terms of the mobility tensors $\mu_{ii}$ are obtained from the $u^{\left(n\right)}\left(R_{n}\right)$ or the $V^{\left(n+1\right)}$ by setting $R_{n}=-R_{1}-R_{2}-\ldots -R_{n-1}$ and suppressing the $V^{\left(1\right)}$. One obtains for the relevant parts of the mobility tensor:

(49) $$b_{12}=-\frac{1}{f_{c}}\frac{9}{8}\frac{R_{h1}R_{h2}R_{g2}^{2}}{R_{1}^{4}}{\hat{R}}_{1}{\hat{R}}_{1}$$

and:

(50) $$b_{123}\cdot V_{1}=u^{\left(3\right)}{\left(r\right)|}_{r\to -R_{1}-R_{2}}.$$

Taking appropriate ensemble averages over these tensors leads to a pseudovirial expansion for the self-diffusion coefficient, viz.,

(51) $$D_{s}\left(c\right)=D_{s0}\left(1-\frac{9}{16}\frac{R_{h1}R_{h2}}{a_{o}R_{g}}\left(\frac{4\pi }{3}R_{g}^{3}\right)c+9.3\cdot 10^{-4}\frac{R_{h1}R_{h2}R_{h3}}{a_{o}R_{g}^{2}}{\left(\frac{4\pi }{3}R_{g}^{3}\right)}^{2}c^{2}+\ldots \right).$$

The numerical coefficient in the $c^{2}$ term was obtained by Monte Carlo integration.

We have now used a generalization of the Kirkwood–Riseman model to treat interchain hydrodynamic interactions. The motions of each chain set up wakes in the surrounding fluid. The surrounding fluid drives the motion of other chains in the fluid, creating fresh wakes which act on still further chains in the sequence. Our generalization has several lacunae. Intrachain hydrodynamics were not included in the calculation. The accuracy of the calculation will diminish when chains overlap, due to the strong interchain hydrodynamic interactions between pairs of nearly adjacent beads.

### Short-Range Hydrodynamic Effects

The purpose of this subsection is to reveal some of the ways in which higher-order hydrodynamic interactions modify polymer dynamics. I follow the results of Phillies and Kirkitelos [35]. There are very considerable opportunities for extending the results of Ref. [35].

Equations (27)–(30) introduce short-range hydrodynamic interactions, corrections to the Oseen tensor approximation that become most important when the diffusing bodies are close together. Consequences of short-range hydrodynamic interactions for the diffusion of colloidal spheres have been intensively studied [77]. Because beads of the same polymer are obliged to remain close to each other, the effects of short-range hydrodynamic interactions are reasonably expected to be at least as important for polymer dynamics as for colloid dynamics. Several authors [12,78,79] have developed multiple scattering approaches for treating polymer–polymer interactions, but none of these developments have included short-range interactions. Freed [80] previously identified the use of short-range hydrodynamic interactions as an unexplored possibility in this context.

Some effects of short-range interactions on polymer diffusion have already been examined. The Oseen tensor $T_{ij}$ effectively approximates the interacting bodies as points, an approximation conspicuously dubious when treating the diffusion of a linear rod polymer around its major axis. Bernal [81] models a rod as a shell of small spheres in order to remove the approximation. The DeWames–Zwanzig singularity [82,83] in the Kirkwood–Riseman [5] treatment of translational diffusion by a rigid rod was shown by Yamakawa [84] to be eliminated by including the $O({\left(a/r\right)}^{3})$ corrections to the Oseen tensor.

Phillies and Kirkitelos [35] made two applications of the short-range hydrodynamic interaction tensors. First, they calculated the chain–chain hydrodynamic interaction tensors including bead–bead interactions out to the $O({\left(a/r\right)}^{7})$ level, both for the chain–chain $T_{ij}$ and to a higher level for the chain–chain $b_{ij}$. They further calculated the effect of the short-range hydrodynamic interactions on the diffusion coefficients of a free monomer and for a monomer bead incorporated into a polymer chain in solution. These effects are entirely distinct from the contribution of short-range hydrodynamic interactions to the chain–chain hydrodynamic interaction tensors. Because the beads of a polymer are always close to other beads of the same chain, at no polymer concentration can the diffusion coefficient of a chain monomer be as large as the diffusion coefficient of a free monomer. At concentrations below the overlap concentration, solvent molecules readily penetrate into polymer coils, but polymer chains do not interpenetrate a great deal. As a result, the addition of polymer molecules to a dilute solution is more effective at delaying the motion of free monomers than at delaying the motion of monomer units of a given polymer chain. At polymer concentrations above the chain overlap concentration, the total polymer concentration is the same everywhere in the solution, but the correlation hole created by a chain of interest ensures that the concentration of the other chains, near the beads of the chain of interest, is never as large as the average concentration of chains in the solution. As a result, the effect of interchain interactions on the mobility of a given polymer bead is never as large as the effect of the same interactions on the mobility of a free monomer in the solution.

Higher-order hydrodynamic interactions make contributions of the same nature to the drag coefficients of a free monomer and a whole chain. However, the contributions to the free monomer and chain drag coefficients are not equal; nor are they multiplicative, contrary to the core assumption behind the common practice of normalizing polymer transport data with small-molecule diffusion coefficient data as a correction for ’monomer friction effects’. The notion that the concentration dependence for $D_{s}$ for free monomers or solvent molecules reveals the concentration dependence of the mobility of monomer units within a polymer chain is therefore incorrect. However, the effect of interchain interactions on the free monomer mobility and on the mobility of monomer units of polymers can be calculated separately.

## 5. Extended Kirkwood–Riseman Model for the Viscosity

This section considers the contribution to the solution viscosity η from chain–chain hydrodynamic interactions, as obtained from an extended Kirkwood–Riseman model. We obtain the lead terms in a pseudovirial expansion for η(c). The underlying hydrodynamic interactions depend on the interchain distance *r* as $r^{-2}$ or $r^{-3}$, so the convergence of the pseudovirial expansion’s cluster integrals is potentially delicate. Our general approach is to apply a velocity field to the solution, and calculate the additional power dissipation caused by the polymer beads as they move with respect to the solvent.

### 5.1. Flow Fields from Scattering of a Shear Field

We choose to impose a spatially oscillatory flow field:

(52) $$u^{\left(0\right)}\left(r\right)=u_{o}\cos\left(kx\right)\hat{j};$$

$u^{\left(0\right)}\left(r\right)$ is the bare velocity field and *k* is the spatial oscillation frequency. The oscillations are not time-dependent, so the shear magnitude is $\left|\alpha \left(x\right)\right|=u_{0}k\sin\left(kx\right)$. The mean-square average shear is $\langle \alpha^{2}\rangle =u_{0}^{2}k^{2}/2$. The shear is assumed to be sufficiently weak that the average spherical symmetry of the polymer chain is not perturbed.

The effect of the spatial oscillations is to ensure that the total of the external forces, applied to the fluid to create the flow field, vanishes. At the end of the calculation, we take the limit k→0. As seen below, the scattering of the velocity field by the polymer molecules makes an additional contribution to the flow field, so that the experimentally measured velocity field will not be the field given by Equation (52). The observable shear field will include the contributions due to the scattering of the imposed shear field by all the polymers in the solution.

The power *P* dissipated by polymer chains in a solution flow is:

(53) $$P=\langle \sum_{i=1}^{M}\sum_{j=1}^{N}f_{ij}{\left(v_{ij}-u\left(r_{ij}\right)\right)}^{2}\rangle .$$

Here, the sum proceeds over all *N* beads of each of the *M* chains in some volume *V*, with $f_{ij}$ being the drag coefficient of bead *j* of chain *i*, $v_{ij}$ being the velocity of that bead, and $u(r_{ij})$ being the velocity that the solvent would have had, at the location $r_{ij}$ of the bead in question, if the bead had been absent.

The viscosity increment is extracted from *P* via the relationship:

(54) $$\frac{dP}{dV}=\delta \eta {\left(\frac{\partial u_{y}}{\partial x}\right)}^{2},$$

where the velocity shear was simplified to correspond to the flow field directions described by Equation (52).

To describe the polymer chains and their motions, we use the same notation as that introduced in the previous section. Because the fluid motions are not the same as in the self-diffusion problem, the calculational details change.

Each chain’s center-of-mass translational velocity is the average of the velocities of its *N* beads, so:

(55) $$V^{\left(i\right)}=\frac{1}{N}\sum_{j=1}^{N}v_{j}.$$

The translational, rotational, and internal mode components of the chain motion are independent of each other, so the rotational velocity vectors $\Omega^{\left(i\right)}$ follow from:

(56) $$\frac{1}{N}\sum_{j=1}^{N}s_{j}\times \left(\Omega^{\left(i\right)}\times s_{j}\right)=\frac{1}{N}\sum_{j=1}^{N}s_{j}\times v_{j}.$$

As in the previous section, the zero-force and zero-torque Equations (35) and (36) determine how each chain moves.

The applied solvent flow within chain n+1 is obtained from $u^{\left(n\right)}$ via a Taylor expansion around the center of mass of chain n+1, to wit:

(57) $$u^{\left(n\right)}\left(R_{n}+s\right)=u^{\left(n\right)}\left(R_{n}\right)+\left(s\cdot \nabla \right)u^{\left(n\right)}\left(R_{n}\right)+\frac{1}{2}{\left(s\cdot \nabla \right)}^{2}u^{\left(n\right)}\left(R_{n}\right)+\ldots$$

The $u^{\left(n\right)}$ are in part determined by $a_{1}$, the location of the first chain, and those of the $R_{j}$ with j<n, these being the displacement vectors taking one from chain 1 to chain *n*.

For the first chain, after making a Taylor series expansion of the fluid velocity around the chain center of mass $a_{1}$ (with $s_{x}=s\cdot i$ and $a_{x}=a_{1}\cdot i$), the zero-force condition may be written:

(58) $$\begin{array}{c} \int ds\;f\left(s\right)g\left(s\right)\left(V^{\left(1\right)}+\Omega^{\left(1\right)}\times s+\dot{w}\left(s\right)-u_{0}\cos\left(ka_{x}\right)\hat{j}\right.\\ \;\;\;\;\;\;\;\;\;\;\;\;\;\;\;\;\;\;\;\;\;\;\;\;\;\;\;\;\;\;\;\;\;\;\;\;\;\;\;\;\;\;\;\;\;\;\;\;\;\;\;\;\;\;\;\;\;\;\;\;\;\;\;\;\;\;\;\left.-\alpha \left(a_{x}\right)s_{x}\hat{j}-\frac{1}{2}{\left(s\cdot \nabla \right)}^{2}u^{\left(0\right)}\left(a_{1}\right)-\ldots \right)=0. \end{array}$$

Because we are discussing weak shear, f(s)g(s) is spherically symmetric, so only terms even in s survive integration, leading to:

(59) $$V^{\left(1\right)}=u_{0}\cos\left(ka_{x}\right)\hat{j}+O\left(s^{2}\right).$$

Up to terms in ${\left(s\cdot \nabla \right)}^{2}$, the first chain simply moves with the velocity that the solvent would have had, at the chain’s center of mass location, if the chain were not present.

Substituting for $v^{\left(1\right)}$ and $u^{\left(0\right)}$, the corresponding zero-torque condition is:

(60) $$\begin{array}{c} \int ds\;f\left(s\right)g\left(s\right)s\times \left(V^{\left(1\right)}+\Omega^{\left(1\right)}\times s+\dot{w}\right)=\\ \;\;\;\;\;\;\;\;\;\;\;\;\;\;\;\;\;\;\;\;\;\;\;\;\int ds\;f\left(s\right)g\left(s\right)[s\times \left(u_{0}\cos\left(ka_{x}\right)\hat{j}+\alpha \left(a_{x}\right)s_{x}\hat{j}+\frac{1}{2}{\left(s\cdot \nabla \right)}^{2}u^{\left(0\right)}\left(s\right)-\ldots \right)]. \end{array}$$

We denote $\int ds\;f\left(s\right)g\left(s\right)Q\left(s\right)=F_{o}\langle Q\left(s\right)\rangle$. Applying an extended series of identities seen in Ref. [45], one finally obtains:

(61) $$\Omega^{\left(1\right)}=\frac{\alpha (a_{x})}{2}\hat{k},$$

which is the result of Kirkwood and Riseman [5] for a single chain in a shear. The chain on the average rotates at half the shear rate at its center of mass.

Chain 1 cannot be stationary at every bead with respect to the fluid. For example, it is doing whole-body rotation, so some of its beads are moving in directions perpendicular to the direction of the fluid flow. The fluid flow, bead velocity, and Oseen tensor then combine to give the fluid flow $u^{\left(1\right)}\left(r\right)$ induced by the first polymer chain, namely:

(62) $$u^{\left(1\right)}\left(r\right)=\int ds\;f\left(s\right)g\left(s\right)T\left(r-s\right)\cdot \left(v^{\left(1\right)}\left(s\right)-u^{\left(0\right)}\left(s\right)\right).$$

A Taylor-series expansion of the Oseen tensor is:

(63) $$T\left(r-s\right)=T\left(r\right)-s\cdot \nabla T\left(r\right)+O\left(s^{2}\right),$$

where:

(64) $$s\cdot \nabla T\left(r\right)=\frac{1}{8\pi \eta_{o}}\left(\frac{s\hat{r}}{r^{2}}+\frac{\hat{r}s}{r^{2}}-\frac{s\cdot \hat{r}}{r^{2}}\left(I+3\hat{r}\hat{r}\right)\right).$$

Upon substituting in Equation (62) for T, $V^{\left(1\right)}$, and $u^{\left(0\right)}$, and applying identities for integrals over s, the induced flow is:

(65) $$u^{\left(1\right)}\left(r\right)=\frac{F_{o}\alpha S^{2}}{8\pi \eta_{o}r^{2}}\frac{xy}{r^{2}}\hat{r}.$$

The process now advances by iteration. $u^{\left(1\right)}\left(r\right)$ acts through a vector $R_{1}$ on chain 2 inducing in it a translational velocity:

(66) $$V^{\left(2\right)}=\frac{F_{o}\alpha S^{2}}{8\pi \eta_{o}R_{1}^{2}}\frac{X_{1}Y_{1}}{R_{1}^{2}}{\hat{R}}_{1},$$

and a rotational velocity:

(67) $$\Omega^{\left(2\right)}=\frac{1}{2}\frac{F_{o}\alpha S^{2}}{8\pi \eta_{o}R_{1}^{3}}\left[\left(\frac{X_{1}^{2}-Y_{1}^{2}}{R_{1}^{2}}\right)\hat{k}+\frac{Y_{1}Z_{1}}{R_{1}^{2}}\hat{j}-\frac{X_{1}Z_{1}}{R_{1}^{2}}\hat{i}\right].$$

Here, $R_{1}\equiv \left(X_{1},Y_{1},Z_{1}\right)$.

The fluid flow that has been double scattered by chains 1 and 2 is:

(68) $$\begin{array}{c} u^{\left(2\right)}\left(R_{1},R_{2}\right)=\\ \;\;\;\;\;\;\;\;\;\;\;\;\;\;\;\;\;+\alpha {\left(\frac{F_{o}S^{2}}{8\pi \eta_{o}}\right)}^{2}\frac{{\hat{R}}_{2}}{R_{1}^{3}R_{2}^{2}}\left[\frac{X_{1}Y_{2}+Y_{1}X_{2}}{R_{1}R_{2}}\left({\hat{R}}_{1}\cdot {\hat{R}}_{2}\right)+\frac{X_{1}Y_{1}}{R_{1}^{2}}\left[1-5{\left({\hat{R}}_{1}\cdot {\hat{R}}_{2}\right)}^{2}\right]\right]. \end{array}$$

Phillies [45] supplies the corresponding large expressions for $V^{\left(3\right)}$, $\Omega^{\left(3\right)}$, and $u^{\left(3\right)}$.

### 5.2. Power Dissipated by Chains in a Shear Field

We now advance to calculate the power dissipated by the polymer molecules as they move with respect to the fluid. The simplest case refers to dilute chains in a shear α, for which Equation (53) becomes:

(69) $$P=\langle M\sum_{i=1}^{N}f_{i}{\left(V^{\left(1\right)}+\frac{\alpha }{2}\hat{k}\times s_{i}+{\dot{w}}_{i}-u^{\left(0\right)}\left(R_{i}\right)-\alpha \left(x\right)s_{x}\hat{j}\right)}^{2}\rangle .$$

$V^{\left(1\right)}$ and $u^{\left(0\right)}\left(R_{1}\right)$ are canceled. In the model, internal chain modes are neglected, so the ${\dot{w}}_{i}$ does not modify the viscosity. Changing variables from $\sum_{i=1}^{N}f_{i}$ to ∫dsf(s)g(s), applying needed identities for the integrals on s, and averaging 〈⋯〉 over chain configurations and positions,

(70) $$P_{1}=N_{c}\frac{F_{o}S^{2}}{6}\alpha^{2}.$$

The average over chain positions is needed because the shear rate depends on the position. In the above calculation, the limit k→0 could have been taken either before or after the positional average.

We calculated above the scattering of the shear field by a specific first chain to a specific second chain, etc. The flow field acting on a given bead includes the original shear field and also all scattered flows that reach that bead. On the same line, the center-of-mass velocity and rotation rate of a given chain are simply the sums of the center-of-mass velocities and rotation rates induced by all flows acting on the given chain.

We now introduce a systematical notation that includes all scattering events. The chain locations are more useful as variables than the displacement vectors. The flow created at r by single scattering from a chain at $a_{2}$ is:

(71) $$u^{\left(1\right)}\left(R_{1}\right)\equiv u^{\left(1\right)}\left(a_{2},r\right).$$

Similarly, the double-scattered flow at r due to beads 2 and 3 is $u^{\left(2\right)}\left(a_{2},a_{3},r\right)$, and so forth.

The total flow field at r due to single scattering of the shear field by all chains other than the representative chain 1 is:

(72) $$u^{\left(1T\right)}\left(r\right)=\sum_{j=2}^{N_{c}}u^{\left(1\right)}\left(a_{j},r\right).$$

For double-scattered flows, a similar notation arises,

(73) $$u^{\left(2T\right)}\left(r\right)=\sum_{\begin{array}{c} j=1\\ k=2\\ j\neq k \end{array}}^{N_{c}}u^{\left(2\right)}\left(a_{j},a_{k},r\right).$$

with the restriction on the double sum being that the last chain in the series cannot be chain 1.

What we next do is to calculate all of the flow fields at the representative chain 1. This includes the original shear field at chain 1, and the flow fields created at chain 1 by each of the other chains in the solution, and the flow fields that were created by one chain and scattered by a second chain before reaching chain 1. We then calculate the power dissipation due to chain 1, averaged over all locations of all chains, calculating the total shear gradient, and finally find the contribution of the representative chain 1 to the viscosity increment.

Chain 1 is a representative chain, it could equally be any chain in the solution. If chain 1 is at r, so $r\equiv a_{1}$, the $u^{\left(1\right)}\left(a_{j},a_{1}\right)$, $u^{\left(2\right)}\left(a_{j},a_{k},a_{1}\right)$,…induce chain motions $V^{\left(2\right)}\left(a_{j},a_{1}\right)$, $\Omega^{\left(3\right)}\left(a_{j},a_{k},a_{1}\right)$, etc., as calculated above. The zeroth-scattering-order velocities $V^{\left(1\right)}\equiv V^{\left(1T\right)}$ and $\Omega^{\left(1\right)}\equiv \Omega^{\left(1T\right)}$ are created by the initial shear field. The higher-order parts of $V^{\left(nT\right)}$ and $\Omega^{\left(nT\right)}$, the parts with n>1, are due to scattering by all combinations of other particles, so:

(74) $$V^{\left(2T\right)}\left(a_{1}\right)=\sum_{j=2}^{N_{c}}V^{\left(2\right)}\left(a_{j},a_{1}\right)$$

and correspondingly:

(75) $$\Omega^{\left(3T\right)}\left(a_{1}\right)=\sum_{\begin{array}{c} j=1\\ k=2\\ j\neq k \end{array}}^{N_{c}}\Omega^{\left(3\right)}\left(a_{j},a_{k},a_{1}\right).$$

In these sums, the neighboring arguments of a $u^{\left(n\right)}$, $V^{\left(n\right)}$, or $\Omega^{\left(n\right)}$ must be distinct.

The total velocity at chain 1 is:

(76) $$V=\sum_{n=1}^{\infty }V^{\left(nT\right)},$$

while for rotation;

(77) $$\Omega =\sum_{n=1}^{\infty }\Omega^{\left(nT\right)}.$$

The Debye form for the power dissipated by a representative chain is obtained from a sum over the *N* beads of the chains:

(78) $$\begin{array}{c} P=\langle \sum_{i=1}^{N}f_{i}\left(V^{\left(1T\right)}+\Omega^{\left(1T\right)}\times s_{i}+V^{\left(2T\right)}+\Omega^{\left(2T\right)}\times s_{i}+\ldots \right.\\ \;\;\;\;\;\;\;\;\;\;\;\;\;\;\;-{\left.u^{\left(0\right)}\left(r_{i}\right)-u^{\left(1T\right)}\left(r_{i}\right)-\ldots \right)}^{2}\rangle . \end{array}$$

We advance with Taylor series expansions in $s_{i}$. As seen above, to lowest order in s, $V^{\left(n+1T\right)}$ and $u^{\left(nT\right)}$ cancel term-by-term for all *n*, so:

(79) $$P=\sum_{i=1}^{N}f_{i}{\left[\Omega^{\left(1T\right)}\times s_{i}+\Omega^{\left(2T\right)}\times s_{i}+\ldots -s_{i}\cdot \nabla u^{\left(0\right)}\left(a_{1}\right)-s_{i}\cdot \nabla u^{\left(1T\right)}\left(a_{1}\right)-\ldots \right]}^{2}.$$

The square generates three sorts of terms. Averaging over chain configurations,

(80) $$\begin{array}{c} \langle \left(s\cdot \nabla \right)u^{\left(n\right)}\cdot \left(s\cdot \nabla \right)u^{\left(m\right)}\rangle \equiv \langle \sum_{\left(i,j,m)=(x,y,z\right)}s_{i}\frac{\partial u_{m}^{\left(a\right)}}{\partial x_{i}}\cdot s_{j}\frac{\partial u_{m}^{\left(b\right)}}{\partial x_{j}}\rangle \\ \;\;\;\;\;\;\;\;\;\;\;\;\;\;\;\;\;=\langle \frac{S^{2}}{3}\sum_{i,m=(x,y,z)}\frac{\partial u_{m}^{\left(a\right)}}{\partial x_{i}}\frac{\partial u_{m}^{\left(b\right)}}{\partial x_{i}}\rangle . \end{array}$$

Terms in $s_{i}s_{j}$ with i≠j average towards zero.

In addition:

(81) $$\langle \left(\Omega^{\left(a\right)}\times s\right)\cdot \left(\Omega^{\left(b\right)}\times s\right)\rangle =\frac{2}{3}S^{2}\Omega^{\left(a\right)}\cdot \Omega^{\left(b\right)}$$

and:

(82) $$\langle \Omega^{\left(a\right)}\times s\cdot \left(s\cdot \nabla \right)u^{\left(b\right)}\rangle =\langle \Omega^{\left(a\right)}\cdot s\times \left(s\cdot \nabla u^{\left(b\right)}\right)\rangle ,$$

while from the zero torque condition:

(83) $$\langle s\times \left(s\cdot \nabla \right)u^{\left(b\right)}\rangle =\frac{2F_{o}S^{2}}{3}\Omega^{\left(b+1\right)}.$$

We obtain the general form for the power dissipation, namely:

(84) $$P=\sum_{a=0}^{\infty }\sum_{b=0}^{\infty }P_{a,b}$$

with:

(85) $$P_{a,b}=\langle \frac{F_{o}S^{2}}{3}\left[\sum_{i=1}^{3}\sum_{j=1}^{3}\left[u_{i,j}^{\left(aT\right)}u_{i,j}^{\left(bT\right)}\right]-2\Omega^{\left(a+1T\right)}\cdot \Omega^{\left(b+1T\right)}\right]\rangle .$$

The Einstein derivative notation:

(86) $$u_{l,j}^{\left(aT\right)}\equiv \left(\partial u^{\left(aT\right)}\cdot \hat{l}/\partial x_{j}\right)$$

(where j,l=1,2,3 represent the three Cartesian coordinates) is in use. The average is over all chain locations. In the first sum, a≠b is allowed. For example, a particle rotating at $\Omega^{\left(2\right)}$ is moving not only with respect to the driving flow $u^{\left(1\right)}$ but also with respect to the original imposed shear field $u^{\left(0\right)}$.

### 5.3. The Total Shear Field

In the previous subsection, the bare shear field was $u_{o}\cos\left(kx\right)\hat{j}$. The polymer motions and the flow fields that they create can all be traced back to the bare shear field and subsequent scattering events. However, if one performed a viscosity measurement, one applies a force, obtains some shear rate, and measures the required force and the corresponding shear field.

We considered fluid flows and power dissipation created by an imposed shear field $du_{y}^{\left(0\right)}/dx=u_{0}\sin\left(kx\right)$. The imposed field created further flows $u^{\left(1\right)}$, $u^{\left(2\right)}$, ⋯ via scattering from the polymers in solution. All flows are part of the total flow $u^{\left(T\right)}$ and its associated shear, $du_{y}^{\left(T\right)}/dx$. Physically, only the total flow can be experimentally measured. The imposed shear is inaccessible to physical observation, so it must be replaced by the total shear. There is here a physical analogy with the replacement made in calculating the dielectric constant, in which the induced dipoles and the total electric field including material contributions must both be calculated, as discussed in this context by Peterson and Fixman [85].

The shear field at (X,Y,Z), due to scattering by a polymer a displacement $-R_{1}$ away, is:

(87) $$\frac{du_{y}^{\left(1\right)}\left(R_{1}\right)}{dx}=\frac{F_{o}S^{2}}{8\pi \eta R_{1}^{3}}\left(\frac{Y_{1}^{2}}{R_{1}^{2}}-\frac{5X_{1}^{2}Y_{1}^{2}}{R_{1}^{4}}\right)u_{0}k\sin\left(k\left(X-X_{1}\right)\right).$$

A similar but more complex form [45] gives the shear transmitted from double scattering through $R_{1}$ and $R_{2}$ to a location (X,Y,Z). An ensemble average over all particle locations, practicable thanks to Mathematica for doing the final integrals, gives the parts of the total shear arising from single and double scattering. For single scattering, one has:

(88) $$\langle \frac{du_{y}^{\left(1\right)}}{dx}\rangle =\frac{16\pi }{15}\frac{F_{0}S^{2}}{8\pi \eta }cu_{0}k\sin\left(kx\right),$$

where *c* is the number density of polymer molecules. For the double-scattered shear,

(89) $$\langle \frac{du_{y}^{\left(2\right)}}{dx}\rangle =-\frac{16\pi^{2}}{75}\frac{F_{0}^{2}S^{4}}{\eta^{2}}c^{2}u_{o}k\sin\left(kx\right)$$

Integrals of $r^{-3}$ over all space do not converge. Because we chose a spatially oscillatory imposed shear field, in preparation for later taking a small-k limit, we obtained convergent integrals for $\langle \frac{du_{y}^{\left(1\right)}}{dx}\rangle$ and $\langle \frac{du_{y}^{\left(2\right)}}{dx}\rangle$, at least when $R_{1}$ and $R_{2}$ are integrated over ranges [a,b], the limits b→∞ and a→0 are then taken. From Equations (52), (88) and (89), we obtain the total shear through second-order concentration contributions, namely:

(90) $$\langle \frac{du_{y}^{\left(T\right)}\left(x\right)}{dx}\rangle =-u_{o}k\sin\left(kx\right)\left[1-\frac{2}{15}\frac{F_{0}S^{2}}{\eta }c+\frac{16\pi^{2}}{75}\frac{F_{0}^{2}S^{4}}{\eta^{2}}c^{2}+O\left(c^{3}\right)\right].$$

### 5.4. Linear and Quadratic Terms—The Huggins Coefficient

We now calculate seriatim the contributions $P_{a,b}$ to the dissipated power, in Equation (84). On dividing out the square of the total shear, Equation (90), a pseudovirial series for the viscosity is obtained.

The lowest-order term in the series is $P_{0,0}$. Combining the results above for $u^{\left(0\right)}$ and $\Omega^{\left(1\right)}$, and taking needed derivatives and integrals:

(91) $$P_{0,0}=\langle N_{c}\frac{F_{o}S^{2}}{3}\left[{\left(u_{o}k\cos\left(ka_{1x}\right)\hat{j}\right)}^{2}-2{\left(\frac{1}{2}u_{o}k\cos\left(ka_{1x}\right)\hat{k}\right)}^{2}\right]\rangle .$$

where 〈⋯〉 is the ensemble average over-chain center-of-mass locations. Including contributions by all $N_{c}$ polymer molecules,

(92) $$P_{0,0}=\frac{N_{c}F_{o}S^{2}}{6}\frac{{\left(u_{o}k\right)}^{2}}{2}.$$

The full power series for *P* is infinite. To evaluate, we must truncate or resume the series. Here, we advance by truncation. There are two obvious choices of truncation variable. Terms could be ordered by the number of scattering events that they include. Terms could also be ordered by how many different particles they include. The lowest-order truncation gives the terms with zero scattering events and one polymer chain; these are the terms analyzed by Kirkwood and Riseman. All higher-order truncations are of mixed order: either they include all terms with a given number of particles but omit some terms involving a given number of scattering events, or alternatively they include all terms involving a given number of scattering events, but omit some terms involving a given number of particles. Higher-order $P_{a,b}$ includes terms that only involve a few chains but incorporate many scattering events, because flow fields can be scattered back and forth between two chains an arbitrary number of times. However, the forms for $u^{\left(1\right)}$, $u^{\left(2\right)}$, and $u^{\left(3\right)}$ show that each scattering event reduces the interaction range by an additional factor of $1/r^{3}$. By analogy with the equilibrium theory of electrolyte solutions, we retain the longest-range interactions, in which a $u^{\left(n\right)}$ couples n+1 distinct chains. These interactions, the ring diagrams, provide the leading terms of $P_{a,b}$. They describe scattering by a series of scattering chains at $a_{2},\ldots ,a_{a}$, finally reaching chain 1 at $a_{1}$. Particle 1 is simply a representative particle; we compute all the scattered flows acting on particle 1, and use them to compute the total power dissipated by chain 1.

The model here leads to a power series in c[η], thus agreeing with the phenomenological observation that [η] is a good reducing variable for *c*. $P_{0,0}$, evaluated above, is proportional to ${\left(c\left[\eta \right]\right)}^{1}$. In $P_{a,b}$, in the factors $u_{i,j}^{\left(aT\right)}u_{i,j}^{\left(bT\right)}$ and $\Omega^{\left(a+1T\right)}\cdot \Omega^{\left(b+1T\right)}$, the chains in the *a* and *b* terms may be the same or may be entirely or partly different. For each independent $a_{j}$, the ensemble average yields a factor $N_{c}$, which is the number of different polymer chains that *j* could have represented. Each chain appearing in one of the $u_{i,j}$ corresponds to a scattering event, each event giving a factor $F_{o}S^{2}/\eta_{o}$. The leading terms of the $P_{a,b}$ are thus ${\left(N_{c}F_{o}S^{2}/\eta_{o}\right)}^{a+b}∼{\left(c\left[\eta \right]\right)}^{a+b}$, so the power series for *P* itself is an expansion in powers of c[η].

At long range, the hydrodynamic interaction tensors describing the $\Omega^{\left(n\right)}$ and $u^{\left(n\right)}$ depend on interparticle spacings as $r^{-3}$. Divergences were avoided because we took a sinusoidal imposed flow $∼u_{o}\cos\left(kx\right)$ and then took the long-wavelength k→0 limit. The hydrodynamic interaction tensors also diverge at short range. We supply an effective short-range cutoff, because the physical $u^{\left(n\right)}$ and $\Omega^{\left(n\right)}$ are finite at small *r*. Peterson and Fixman [85] proposed a related cutoff, namely that two overlapped chains were approximated as moving as a rigid dumbbell.

We now compute the $O(c^{2})$ contributions to η, these being the $P_{a,b}$ with a+b=1 or a=b=1. Terms with two chains and more scattering events are allowed by the formalism but will be smaller because the interactions will be shorter-ranged. For a+b=1:

$$P_{1,0}=P_{0,1}=\int da_{1}\;da_{2}\;\ldots \;da_{N_{c}}\;\exp(-\beta \left(W_{N_{c}}-A_{N_{c}}\right)\times$$

(93) $$\left[\sum_{p\neq q=1}^{N_{c}}\frac{F_{o}S^{2}}{3}\left(-2\Omega^{\left(1\right)}\left(a_{p}\right)\cdot \Omega^{\left(2\right)}\left(a_{q},a_{p}\right)+\sum_{i,j=1}^{3}\left[u_{i,j}^{\left(0\right)}\left(a_{p}\right)u_{i,j}^{\left(1\right)}\left(a_{q},a_{p}\right)\right]\right)\right].$$

Here, $k_{B}$ is Boltzmann’s constant, $\beta ={\left(k_{B}T\right)}^{-1}$, *T* is the absolute temperature, $W_{N_{c}}$ is the potential energy, $A_{N_{c}}$ is the normalizing factor, and the *p* and *q* label chains. The average over internal chain coordinates gives an $S^{2}$.

All terms of the sum over *p* and *q* are identical save for the label. The ensemble average is:

$$P_{1,0}=\frac{F_{o}S^{2}N_{c}\left(N_{c}-1\right)}{3}\int da_{1}\;da_{2}\;\left[\left(\sum_{i,j=1}^{3}\left[u_{i,j}^{\left(0\right)}\left(a_{1}\right)u_{i,j}^{\left(1\right)}\left(a_{2},a_{1}\right)\right]\right.\right.$$

(94) $$\left.\left.-2\Omega^{\left(1\right)}\left(a_{1}\right)\cdot \Omega^{\left(2\right)}\left(a_{2},a_{1}\right)\right)\int da_{3}\;\ldots da_{M}\;\exp(-\beta \left(W_{M}-A_{M}\right)\right].$$

The non-zero derivative of $u^{\left(0\right)}$ is:

(95) $$u_{,x}^{\left(0\right)}=-u_{o}k\sin\left(ka_{1x}\right)\hat{j}$$

where $a_{1x}$ is the *x* component of $a_{1}$. The matching derivative of $u^{\left(1\right)}$ is:

(96) $$u_{,x}^{\left(1\right)}=u_{o}k\sin\left(k\left(a_{1x}-X_{1}\right)\right)\frac{F_{o}S^{2}}{8\pi \eta_{o}}\left[\left(\frac{Y_{1}}{R_{1}^{4}}-\frac{5X_{1}^{2}Y_{1}}{R_{1}^{6}}\right){\hat{R}}_{1}+\frac{X_{1}Y_{1}}{R_{1}^{5}}\hat{i}\right].$$

$a_{1x}$ refers to the final particle in the scattering sequence; $R_{1}\equiv \left(X_{1},Y_{1},Z_{1}\right)$ points from the penultimate to the ultimate particle of the scattering sequence.

The angular velocities appear in Equations (61) and (67). In these equations, α is the shear at the first particle of the scattering series, namely $-u_{o}k\sin\left(ka_{1x}\right)\hat{j}$ and $u_{o}k\sin\left(k\left(a_{1x}-X_{1}\right)\right)\hat{j}$, respectively. The identity $\sin\left(ka_{1x}\right)\sin\left(k\left(a_{1x}-X_{1}\right)\right)=\left(-\cos\left(2ka_{1x}-kX_{1}\right)+\cos\left(kX_{1}\right)\right)/2$ is then applied. The ensemble average only depends on $a_{1}$ through $\cos(2ka_{1x}-kX_{1})$, which vanishes on averaging over $a_{1}$.

Recalling the standard form:

(97) $$\frac{g^{\left(2\right)}\left(r\right)}{V^{2}}=\frac{\int da_{3}\;\ldots \;da_{M}\;\exp\left(-\beta W\left(r,a_{3},\ldots a_{M}\right)\right)}{\int da_{1}\;\ldots \;da_{M_{c}}\exp\left(-\beta W\left(r,a_{3},\ldots a_{M_{c}}\right)\right)}$$

for the radial distribution function, here with $r=a_{2}-a_{1}$,

(98) $$P_{1,0}=-\left(\frac{u_{o}^{2}k^{2}}{2}\right)\left(\frac{N_{c}\left(N_{c}-1\right){\left(F_{o}S^{2}\right)}^{2}}{24\pi \eta_{o}V}\right)\int dR\;g^{\left(2\right)}\left(R\right)\frac{\cos(kX)}{R^{3}}\left[\frac{X^{2}+Y^{2}}{R^{2}}-\frac{10X^{2}Y^{2}}{R^{4}}\right].$$

In the radial integral, the lower cutoff is not required for $P_{1,0}$. Without the cos(kx), the ∫dR diverges at a large *R*; the angular integral vanishes; and the ∫dR is improper. The proper long-wavelength limit results from taking ∫dR and then taking k→0. If the shear were linear and not oscillatory in space, $P_{1,0}$ would be undefined, as observed three-quarters of a century ago by Saito [10].

Choosing k to be parallel to the X axis, a useful identity is [86]:

(99) $$\cos\left(k\cdot R\right)=4\pi \sum_{l=0}^{\infty }\frac{i^{l}+{\left(-i\right)}^{l}}{2}j_{l}\left(kr\right){\left(4\pi \left(2l+1\right)\right)}^{1/2}Y_{l0}\left(\theta \right).$$

Here, $j_{l}$ is a spherical Bessel function, and θ is the angle between k and R.

On invoking spherical coordinates, recourse to Mathematica gives:

(100) $$P_{1,0}=-\eta_{o}\frac{N_{c}^{2}-N_{c}}{V}\frac{48\pi }{5}{\left(\frac{F_{o}S^{2}}{6\eta_{o}}\right)}^{2}\left(\frac{u_{o}^{2}k^{2}}{2}\right).$$

How can this term be negative? Mathematically, in the intrinsically positive form ${\left(a-b\right)}^{2}$ the term −2ab can be negative; in the calculation here, $P_{1,0}$ can play the role of a −2ab. Physically, Equation (100) is negative because $u^{\left(1\right)}$ causes chain 1 to rotate, thereby reducing the velocity difference between chain 1’s beads’ velocities and $u^{\left(0\right)}$, so dissipation is reduced by this term.

We now turn to $P_{1,1}$. Writing $\Omega^{\left(2T\right)}$ and $u^{\left(1T\right)}$ as sums over all the other particles in the system,

(101) $$\begin{array}{c} P_{1,1}=\langle \frac{N_{c}F_{o}S^{2}}{3}\left(-2\sum_{p,q=2}^{N_{c}}\Omega^{\left(2\right)}\left(a_{p},a_{1}\right)\cdot \Omega^{\left(2\right)}\left(a_{q},a_{1}\right)+\right.\\ \;\;\;\;\;\;\;\;\;\;\;\;\;\;\;\;\left.\sum_{p,q=2}^{N_{c}}\sum_{i,j=1}^{3}\left[u_{j,i}^{\left(1\right)}\left(a_{p},a_{1}\right)u_{j,i}^{\left(1\right)}\left(a_{q},a_{1}\right)\right]\right)\rangle . \end{array}$$

Only the self (p=q) terms of Equation (101) are significant; the distinct (p≠q) terms give an effect cubic in concentration. To $O(c^{2})$:

$$P_{1,1}=\frac{N_{c}\left(N_{c}-1\right)F_{o}S^{2}}{3V}\int da_{1}\;da_{2}\;g^{\left(2\right)}\left(a_{1},a_{2}\right)\left(\Omega^{\left(2\right)}\left(a_{2},a_{1}\right)\cdot \Omega^{\left(2\right)}\left(a_{2},a_{1}\right)\right.$$

(102) $$\left.+\sum_{i,j=1}^{3}\left[u_{j,i}^{\left(1\right)}\left(a_{2},a_{1}\right)u_{j,i}^{\left(1\right)}\left(a_{2},a_{1}\right)\right]\right)+\ldots$$

The convergence here at large *R* is sufficiently strong that the integrals and the k→0 limit can be exchanged, giving:

(103) $$\begin{array}{c} P_{1,1,s}=\frac{c^{2}VF_{o}S^{2}}{6}\alpha^{2}{\left(\frac{F_{o}S^{2}}{8\pi \eta_{o}}\right)}^{2}\times \\ \;\;\;\;\;\;\;\int_{V}dR\;\frac{1}{R^{6}}\left[\frac{6X^{2}Y^{2}-X^{4}-Y^{4}+Z^{4}}{R^{4}}+\frac{2X^{2}+2Y^{2}-Z^{2}}{R^{2}}\right]g^{\left(2\right)}\left(R\right). \end{array}$$

$P_{1,1,s}$ requires a short-range cutoff *a* for the convergence of $\int_{V}dR$. Such a cutoff is physically appropriate. Equations (65) and (67) represent the long-range parts of series expansions. Short-range terms that prevent divergence are represented herein by the cutoff distance. Inserting such a cutoff into $P_{1,0}$ has little effect.

Via integration, one obtains:

(104) $$P_{1,1,s}={\left(\frac{F_{o}S^{2}}{8\pi \eta }\right)}^{2}\frac{4\pi F_{o}S^{2}}{15a^{3}}c^{2}\frac{{\left(u_{o}k\right)}^{2}}{2}.$$

Combining Equations (90), (92), (100) and (104),

$$\eta {\left(\langle \frac{du_{y}^{\left(T\right)}}{dx}\rangle \right)}^{2}=\eta_{o}\left[1+\frac{F_{o}S^{2}}{6\eta_{o}}c+\left(-\frac{4\pi F_{o}^{2}S^{4}}{15\eta_{o}^{2}}+\frac{F_{o}^{3}S^{6}}{240\pi \eta_{o}^{3}a^{3}}\right)c^{2}\right]\times$$

(105) $${\left[1-\frac{2}{15}\frac{F_{0}S^{2}}{\eta }c+\frac{16\pi^{2}}{75}\frac{F_{0}^{2}S^{4}}{\eta^{2}}c^{2}\right]}^{-2}{\left(\langle \frac{du_{y}^{\left(T\right)}\left(x\right)}{dx}\rangle \right)}^{2}.$$

In terms of the series $k_{H}$ of:

(106) $$\eta /\eta_{o}=1+\left[\eta \right]c+k_{H}{\left[\eta \right]}^{2}c^{2},$$

the Huggins coefficient being $k_{H}$,

(107) $$\left[\eta \right]=\frac{13F_{o}S^{2}}{30\eta_{o}}$$

and:

(108) $$k_{H}=\frac{88-240\pi -384\pi^{2}}{169}+\frac{225[\eta ]}{4394\pi a^{3}}.$$

The cutoff radius *a* is a crude approximation. A sound treatment of hydrodynamics of interpenetrated random coils is needed. One reasonably expects *a* to be moderately smaller than *S*.

## 6. From Pseudovirial Series to Higher Concentrations

The above discussion shows how power series expansions may be used to determine the concentration dependence of $D_{s}$ and η. The series approaches face the challenge that at elevated concentrations more and more terms are needed in order to obtain accurate predictions, while at the same time, the scale of the calculations required to obtain additional forms becomes larger and larger. To overcome this difficulty, alternative approaches to computing $D_{s}\left(c\right)$ and η(c) at large *c* have been employed. We here discuss two, namely *self-similarity* and *the Altenberger–Dahler positive-function renormalization group*. Self-similarity advances by physical arguments about chain–chain interactions. The positive-function renormalization group approach proposes to advance by noting that $D_{s}\left(c\right)$ and η(c) both depend on concentration *c* and on a coupling parameter *R*, and their values at large *c* and some *R* are equal to their values at a smaller *c* and some other value of *R*, the values of $D_{s}\left(c\right)$ and η(c) being easier to compute at the smaller *c* and some other *R*. The positive-function renormalization group advances by calculating the needed “other” *R*.

### 6.1. Self-Similarity Approach

This subsection considers the original [23] self-similarity derivation of the universal scaling equation for polymer self-diffusion. The derivation has several basic assumptions. First, at all concentrations, the dominant polymer–polymer interactions are taken to be hydrodynamic, with chain crossing constraints provided at most secondary corrections. The interchain hydrodynamic interactions are approximated as being the same, except for numerical coefficients, as the hydrodynamic interactions between hard spheres. Second, the effects of sequential infinitesimal concentration increments on $D_{s}$ are said to be self-similar, whencefrom the name of the derivation. Third, polymer chains in good solvents are taken to contract as polymer concentration is increased.

The form of the hydrodynamic interactions between polymer chains has been calculated above. A velocity $V^{\left(1\right)}$ of the first polymer chain in a sequence creates a flow field $u^{\left(1\right)}$ in the solvent. The flow field acts on chain 2. The translation and rotation of chain 2 create a further flow field $u^{\left(2\right)}$ and so forth. At every step after chain 2, the final flow field $u^{\left(f\right)}$ can act back on chain 1, inducing in chain 1 an additional translational velocity $\delta V=u^{\left(f\right)}$, with $u^{\left(f\right)}$ as evaluated at chain 1. Take $f_{\mathrm{ch}}=6\pi \eta R_{h}$ to be the drag coefficient of the first chain. The force the first chain would apply to the solvent, if it moved relative to a quiescent solution, is $f_{\mathrm{ch}}^{o}V^{\left(1\right)}$. Multiplying through the entire calculation by $f_{\mathrm{ch}}^{o}$, the force the final flow field would exert back on chain 1 in response to chain 1’s motions is $f_{\mathrm{ch}}^{o}V^{\left(f\right)}$. In Brownian motion, no forces external to the polymer–solvent system act on the polymer chains. The chains move because hydrodynamic fluctuations create flows in the solvent, the chains being moved by the fluctuations, but the fluctuation–dissipation theorem requires that the correlations in the displacements arising from the hydrodynamic fluctuations must be the same as the correlations in the displacements that would appear if chain 1 was subject to an external force that moved chain 1 in the same way with respect to the solvent.

The self-diffusion coefficient of a polymer is determined by its drag coefficient $f_{\mathrm{ch}}$ via the Einstein equation $D_{s}=k_{B}T/f_{\mathrm{ch}}$. $f_{\mathrm{ch}}$ differs from $f_{\mathrm{ch}}^{o}$ in that it includes contributions to the hydrodynamic drag on a chain due to the chain’s interactions with other chains. To determine the concentration dependence of $D_{s}$, it is sufficient to determine the concentration dependence of $f_{\mathrm{ch}}$.

The ability of chain 2 to affect the drag coefficient of chain 1 is determined by the strength of chain 2’s hydrodynamic interactions with the solvent, here approximated by the drag coefficient $f_{\mathrm{ch}}$ and by the coupling coefficient α describing the strength of interchain interactions. We advance by considering the effect of successive infinitesimal concentration increments on $f_{\mathrm{ch}}$. The first increment δc gives us:

(109) $$f_{\mathrm{ch}}\left(\delta c\right)=f_{\mathrm{ch}}\left(0\right)+\alpha f_{\mathrm{ch}}\left(0\right)\delta c=f_{\mathrm{ch}}\left(0\right)\left(1+\alpha \delta c\right).$$

Here, we have applied the approximation that the change in $f_{\mathrm{ch}}$ due to the first concentration increment is proportional to $f_{\mathrm{ch}}$ of the chains in the increment. We now apply a second infinitesimal concentration increment δc. The *self-similarity* step is to assert that the chains of the second concentration increment affect not only the chain of interest but also equally the chains of the first concentration increment, so that:

(110) $$f_{\mathrm{ch}}\left(2\delta c\right)=f_{\mathrm{ch}}\left(0\right)+\alpha f_{\mathrm{ch}}\left(0\right)\delta c+\alpha f_{\mathrm{ch}}\left(\delta c\right)\delta c.$$

On the right-hand-side of the equation, the first two terms are $f_{\mathrm{ch}}\left(\delta c\right)$. The third term is the effect of the second concentration increment δc, written in terms of the drag coefficient $f_{\mathrm{ch}}\left(\delta c\right)$ of the chain at concentration δc. Moving the first two terms from the rhs to the lhs of the equation and dividing by $f_{\mathrm{ch}}\delta c$, one finds:

(111) $$\frac{f_{\mathrm{ch}}\left(2\delta c\right)-f_{\mathrm{ch}}\left(\delta c\right)}{f_{\mathrm{ch}}\left(\delta c\right)\delta c}=\alpha .$$

In the limit δc→0, the left side is recognized as the logarithmic derivative of $f_{\mathrm{ch}}\left(c\right)$, so the integration gives:

(112) $$f_{\mathrm{ch}}\left(c\right)=f_{\mathrm{ch}}\left(0\right)\exp\left[\int_{0}^{c}dc\;\alpha \left(c\right)\right].$$

and correspondingly:

(113) $$D_{s}\left(c\right)=D_{s}\left(0\right)\exp\left[-\int_{0}^{c}dc\;\alpha \left(c\right)\right].$$

At the time of the original derivation of the hydrodynamic scaling model [23] on the basis of self-similarity, the chain–chain hydrodynamic interaction tensors seen above had not yet been obtained. It was instead proposed that Equations (25) and (27), which describe the mobility $\mu_{ii}$ for the pairs of hard spheres, are dimensionally correct for chains, even though they do not supply precise numerical coefficients, and are therefore good as a first approximation to the chain–chain hydrodynamic interaction tensors. The conclusion was that:

(114) $$\alpha \left(c\right)=QR_{h1}R_{g2}^{3}.$$

Here, *Q* includes numerical coefficients and the average of ${\hat{r}}_{ij}{\hat{r}}_{ij}/r_{ij}^{4}$ over the chain–chain radial distribution function, while the sum over spheres in Equation (25) becomes the ∫dc of Equation (113).

The final approximation was to estimate the concentration dependence of the chain radii from the results of Daoud et al. [57]. In the original calculation [23], the radii were taken to scale as:

(115) $$R^{2}∼Mc^{-x},$$

with x=1/4. The original prediction only referred to long chains with *c* greater than some overlap concentration $c^{*}$. For long chains at lower concentrations, the degree of chain contraction was predicted to be less. For short chains, the Daoud et al. model predicts x≈0. Combining the above three equations, one finds the prediction:

(116) $$D_{s}\left(c\right)=D_{0}\exp\left(-Q^{\prime }Mc^{1-2x}\right).$$

$Q^{\prime }$ includes *Q* and other numerical coefficients arising from the integration. Comparing with the universal scaling Equation (5), if one identifies 1−2x=ν, one predicts:

(a) For large polymer chains, ν=0.5, except perhaps at very low concentrations.
(b) For short polymer chains at all concentrations, ν=1.0.
(c) For the probe diffusion coefficient $D_{p}$, the radius $R_{h1}$ of the probe does not depend on the concentration, so ν=1−3x/2≈5/8.

Finally, identifying α of Equation (5) with $Q^{\prime }M$, one predicts $\alpha ∼M^{\delta }$ for δ=1.0. As discussed below, all of the above predictions have been experimentally confirmed.

### 6.2. Positive-Function Renormalization Group

This subsection develops the mathematical structure of the Altenberger–Dahler positive-function renormalization group (PFRG) approach [49,50,51,52,53]. In Section 7, the approach is directly applied to treat the self-diffusion coefficient and the low-shear viscosity. In Section 8, a fixed-point structure for the viscosity is inferred and then applied via an ansatz to infer the frequency dependencies of the loss and storage moduli.

Altenberger and Dahler noted that renormalization group methods have been invoked in several branches of physics to deal with superficially different mathematical challenges. Renormalization group methods were inserted into high-energy theory to cope with the difficulties arising from cutoff wavelengths and the presence of infinities in series expansions. Renormalization group methods appear in statistical mechanics in the applications of self-similarity methods, such as block renormalization, where the methods are used to eliminate insignificant fine details from the descriptions of critical fluctuations. Of more significance here, renormalization group methods can be used to extend the range of validity of lower-order power series expansions. The effort here pursues the last of these uses. We are not facing divergences or systems with a multiplicity of unimportant short-range length scales. We have on hand a low-order power-series expansion that would be inordinately tedious to extend to a very high order.

Because the Altenberger–Dahler PFRG method has not been used extensively, we first sketch the physical rationales that lead to the method and then consider the mathematical forms. The starting point is that many physical properties of a solution can be written as a pseudovirial expansion, e.g.,

(117) $$A\left(c\right)=a_{o}+a_{1}c^{\prime }+a_{2}c^{\prime 2}.$$

Here, *A* is the physical property, $c^{\prime }$ is the solute concentration in physical units, and the $a_{i}$ are the pseudovirial coefficients. The $a_{i}$ are typically obtained from cluster expansions. It is not claimed—that would be incorrect—that all concentration-dependent physical properties have pseudovirial expansions. *A* is actually a function of two parameters, namely the concentration $c^{\prime }$ and a coupling parameter *R*, so one may write $A=A(c^{\prime },R)$. The coupling parameter *R* determines the values of the $a_{i}$. Cases in which there are multiple coupling parameters are included by treating *R* as a vector. The c→0 limit of *A* is simply $a_{o}$. The limit of noninteracting solute molecules can also be obtained as R→0, in which case, once again, $A=a_{o}$. The introduction of a reference concentration $c_{r}$ and dimensionless concentration units $c=c^{\prime }/c_{r}$ leads to:

(118) $$A\left(c\right)=a_{o}+\left[a_{1}c_{r}\right]c+\left[a_{2}c_{r}^{2}\right]c^{2}.$$

At elevated concentrations, the above pseudovirial series become inaccurate. The familiar virial approach is to improve the accuracy of the series by adding additional terms $a_{3}$, $a_{4}$, etc. In the PFRG approach, the series of Equation (118) is taken to be exact, but the bare coupling parameter *R* is replaced with a dressed, concentration-dependent coupling parameter $\bar{R}\left(R,c\right)$. The values of $\bar{R}$ are chosen so that the $a_{i}$ calculated using $\bar{R}$, when inserted into Equation (118), give the correct values for *A* even at large concentrations.

The Altenberger–Dahler calculation has two major parts. First, constraints on the behavior of *A* are used to determine functional requirements for the dressed coupling parameter $\bar{R}\left(R,c\right)$. Second, at low concentrations, $\bar{R}=R$ to high precision. A group of Lie differential equations and infinitesimal generators for the dependencies of *A* and $\bar{R}$ on *c* are then determined by the group properties of $\bar{R}$. The polymer calculation has three further parts. First, the multichain Kirkwood–Riseman model described above is used to obtain the actual $a_{i}$, including the dependencies of the $a_{i}$ on *R*. These dependencies determine Lie group generators and equations needed to compute $\bar{R}$ and *A* for $D_{s}$ or η. For an object of fixed *R*, numerical integration determines A(c) (here, either $D_{s}\left(c\right)$ or η(c)) at the level of precision of the input calculations. Finally, applying the results of Daoud et al. [57] and King et al. [58], on chain contraction at elevated polymer concentration, one obtains an approximation to the universal scaling equation for polymer self-diffusion. The approximation is valid to a specific order in the dressed coupling parameter $\bar{R}$.

To open the renormalization group calculation, the constraint on *A* is that it is positive definite, never zero or negative, so *A* may always be written in the form A=exp(B). It is convenient to transform $A(c^{\prime },R)$ into dimensionless units by normalizing with respect to *A* at some non-zero concentration $c_{o}$, namely:

(119) $$\bar{A}\left(c^{\prime },R\right)=A\left(c^{\prime },R\right)/A\left(c_{o},R\right).$$

Here, $\bar{A}\left(c^{\prime },R\right)$ is the normalized and hence dimensionless transformation of $A(c^{\prime },R)$. $c_{o}$ and $c_{r}$ are independent, but for simplicity, we will choose $c_{o}=c_{r}$ in the following. Because $\bar{A}$ is also positive definite, it may be written as:

(120) $$\bar{A}\left(c^{\prime },R\right)=\exp(\int_{c_{o}}^{c^{\prime }}ds\;L\left(s,R)\right),$$

where:

(121) $$L\left(s,R\right)=\frac{\partial \ln(\bar{A}\left(s,R\right))}{\partial s}.$$

Equations (120) and (121) are an identity. They enforce, and are valid because of, the requirement that $\bar{A}\left(c^{\prime },R\right)$ is positive definite.

In the integral of Equation (120), we introduce an intermediate concentration $z^{\prime }$, and divide the one integral into two, giving:

(122) $$\bar{A}\left(c^{\prime },R\right)=\left(\exp(\int_{c_{o}}^{z^{\prime }}ds\;L\left(s,R\right)\right)\left(\exp(\int_{z^{\prime }}^{c^{\prime }}ds\;L\left(s,R\right)\right).$$

We now go to dimensionless concentration units, choosing $c_{o}$ as a reference concentration with $c=c^{\prime }/c_{o}$, and make a change of variables s→yz, finding:

(123) $$\bar{A}\left(c,R\right)=\bar{A}\left(z,R\right){\left[\exp\left(\int_{1}^{c/z}dy\;L\left(yz,R\right)\right)\right]}^{z}.$$

Because $z^{\prime }$ (in physical units) is intermediate between $c_{o}$ and $c^{\prime }$, *z* (dimensionless units) must be ≥1. Here, $\exp\left(az\right)={\left(\exp\left(a\right)\right)}^{z}$ has been applied. The above equation supports the introduction of a dressed coupling parameter $\bar{R}$. The dressed coupling parameter is chosen so that, at each *z* and *R*,

(124) $$L\left(yz,R\right)=L(y,\bar{R}\left(z,R\right)),$$

so that L at an elevated concentration yz can be replaced by L at a lower concentration *y* by replacing *R* with the appropriate $\bar{R}$. There is an implicit assumption that such an $\bar{R}$ exists. A representative contrary outcome would be that L(y,R) saturates with changes in *R*, so that there is no value of $\bar{R}$ that satisfies Equation (124). This issue does not arise for the calculation here, but should be kept in mind as a general possibility. Replacing *R* with $\bar{R}$ has an analogy in the direct self-similarity calculation of $D_{s}$, namely in those calculations where each chain’s bare drag coefficient $f_{o}$ was replaced with a dressed drag coefficient *f* of the chain at the concentration of interest.

On applying Equation (124) to Equation (123), and applying exp(ln(A))=A, one has:

(125) $$\frac{\bar{A}\left(c,R\right)}{\bar{A}\left(z,R\right)}={\left[\bar{A}\left(\frac{c}{z},\bar{R}\left(z,R\right)\right)\right]}^{z}.$$

In order for this equation to be correct, we must be working in dimensionless units, so that the lower bound of the integral in Equation (123) is unity. Equation (125) represents a numerical renormalization of $\bar{A}\left(c,R\right)$, in that $\bar{A}\left(c,R\right)/\bar{A}\left(z,R\right)=1$ if c=z. Equation (125) also represents a group property, namely showing how the effect on $\bar{A}$ of a change in the concentration *c* can be replaced with a different change in the concentration *c* together with a corresponding dressed coupling parameter $\bar{R}$.

Multiplying Equation (125) by $\bar{A}\left(z,R\right)$, and adopting a new concentration variable via c→cz, one has:

(126) $$\bar{A}\left(cz,R\right)=\bar{A}\left(z,R\right)\left[\bar{A}\left(c,\bar{R}\left(z,R\right)\right)\right]$$

The form of the left-hand-side of the equation forces the right-hand-side of the equation to be symmetric under the interchange of variables *c* and *z*. In consequence, severe constraints are placed on the possible functional forms for $\bar{R}$. In particular, as shown by Altenberger and Dahler [50], in Appendix 1 of their paper, Equation (126) forces the requirement:

(127) $$\bar{R}\left(c,R\right)=\bar{R}\left(c/z,\bar{R}\left(z,R\right)\right).$$

We have now finished the first part of the derivation. We made two assumptions, the first being that $\bar{A}$ as a variable is never ≤0, and the second being that there is an effective coupling parameter $\bar{R}$ that is consistent with Equation (125).

In the second part of the derivation, we show that Equations (125) and (127) lead to differential equations for $\bar{R}$. Note that, at the reference concentration $c_{o}$, one has $\bar{R}\left(c_{o},R\right)=\bar{R}\left(1,R\right)=R$, which gives the boundary condition for integrating the differential equations we are about to obtain. The differential equations are obtained from Equations (125) and (127), beginning by taking derivatives with respect to *c*. From the derivative of Equation (125), one sets c=z and notes $\bar{A}\left(1,\bar{R}\left(z,R\right)\right)=1$ (directly following from Equation (125)), leading for u=c/z to:

(128) $$\frac{\partial \ln(\bar{A}\left(z,R\right))}{\partial z}={\left.\frac{\partial \bar{A}\left(u,\bar{R}\left(z,R\right)\right)}{\partial u}\right|}_{u=1}=\gamma \left(\bar{R}\left(z,R\right)\right).$$

$\gamma (\bar{R}\left(z,R\right))$ is a differential generator. At z=1, the generator becomes:

(129) $${\left.\frac{\partial \bar{A}\left(z,R\right)}{\partial z}\right|}_{z=1}=\gamma \left(R\right).$$

From the derivative of Equation (127) with respect to *c*, upon setting c=z, one obtains:

(130) $$\frac{\partial \bar{R}\left(z,R\right)}{\partial \ln(z)}={\left.\frac{\partial \bar{R}\left(u,\bar{R}\left(z,R\right)\right)}{\partial u}\right|}_{u=1}=\beta \left(\bar{R}\left(z,R\right)\right)$$

as the definition of β. Alternatively, the definition in Equation (130) can be obtained from Equation (128) by taking a derivative of γ with respect to *z*, leading to:

(131) $$\frac{\partial \bar{R}\left(z,R\right)}{\partial z}=\frac{\left({\bar{A}}^{\prime \prime }\left(z,R\right)\right)/\bar{A}\left(z,R\right)-{\left({\bar{A}}^{\prime }\left(z,R\right)/\bar{A}\left(z,R\right)\right)}^{2}}{\partial \gamma (\bar{R}\left(z,R\right)/\partial \bar{R}\left(z,R\right))}.$$

Here, ${\bar{A}}^{\prime }\left(z,R\right)=\partial \bar{A}\left(z,R\right)/\partial z$ and ${\bar{A}}^{\prime \prime }\left(z,R\right)=\partial^{2}\bar{A}\left(z,R\right)/\partial z^{2}$. On setting z=1, a further result for β is obtained from the above two equations, namely:

(132) $$\beta \left(R\right)=\frac{{\bar{A}}^{\prime \prime }\left(1,R\right)/\bar{A}\left(1,R\right)-{\left({\bar{A}}^{\prime }\left(1,R\right)/\bar{A}\left(1,R\right)\right)}^{2}}{\left(\partial \gamma \left(R\right)/\partial \bar{R}\right)}.$$

This final equation gives β as a function of *R* rather than $\bar{R}$, at least at the initial concentration. $\beta (\bar{R})$ and $\gamma (\bar{R})$ provide the infinitesimal generators for Lie equations for the concentration dependencies of $\bar{A}$ and $\bar{R}$.

A variety of methods for integrating these equations are available. The calculation requires as inputs $\bar{A}$ and its derivatives evaluated at z=1. Altenberger and Dahler [49,50] proceed by approximating $\bar{A}$ with its low-order series expansion. For reasonable choices of the initial concentration (in physical units) $c_{o}$, this approximation is not very demanding. Indeed, Altenberger and Dahler use a cubic approximation for *P* of a hard sphere gas, choose an initial volume fraction $c_{o}=0.16$, and obtain the *P* predicted by an eight-term virial fraction for *c* up to 0.62.

## 7. From Renormalization Group to Universal Scaling

In this section, we advance from the hydrodynamic calculations of Section 4 and Section 5 and the positive-function renormalization group approach developed in Section 6.2 to extrapolate the concentration dependence of $D_{s}$ and η. We invoke the Altenberger–Dahler positive-function renormalization group and Equation (51) for the concentration and chain radius dependencies of $D_{s}$ to extrapolate $D_{s}\left(c\right)$ to larger concentrations. Equation (51) includes both $R_{h}$ and $R_{g}$; these are approximated as being a single radius $R^{\prime }$. We identify the concentration variable of the renormalization group calculation as the physical concentration *c*, and choose $R=R^{\prime }/R_{o}$ as the dimensionless coupling parameter. $R_{o}$ is identified as $R^{\prime }$ at c=1.

Equation (51) is now:

(133) $$D_{s}\left(c\right)=D_{o}\left(1+\bar{a}R^{4}c+\bar{b}R^{7}c^{2}\right)$$

All dependence on *R* is now explicit. The renormalized pseudovirial coefficients are:

(134) $$\bar{a}=-\frac{9}{16}\frac{4\pi }{3a_{o}}R_{o}^{4}c_{r}$$

and:

(135) $$\bar{b}=-\frac{9.3\cdot 10^{-4}}{a_{o}}{\left(\frac{4\pi }{3}\right)}^{2}R_{o}^{7}c_{r}^{2}.$$

At concentration $c_{r}$, c=R=1, and *c* and *R* are both dimensionless. These equations differ from the expressions employed by Altenberger and Dahler [49,50] in one significant way. In the earlier calculations, *c* and *R* always appeared as the product cR, so that the $n^{\mathrm{th}}$ term of their virial expansion depended on *R* as $R^{n}$. Here, the *c* and *R* dependencies are distinct.

$D_{s}$ is transformed into $\bar{D_{s}}$ by dividing by $D_{s}\left(1\right)=D_{o}\left(1+\bar{a}R^{4}+\bar{b}R^{7}\right)$. $\bar{A}$ of the prior section is identified as $\bar{D_{s}}$. All dependence of $\bar{D_{s}}$ on *R* can be moved to the numerator via the expansion ${\left(1-x\right)}^{-1}\to 1+x+x^{2}+\ldots$. So long as one truncates at $R^{7}$, which is the highest-order limit of the original hydrodynamic series, one finds:

(136) $$\bar{D_{s}}\left(c\right)=1+\bar{a}R^{4}\left(c-1\right)+\bar{b}R^{7}\left(c^{2}-1\right).$$

Identifying the concentration variable *z* of the prior section with *c* here, γ(R) arises from the logarithmic derivative of $\bar{D_{s}}\left(c\right)$ as:

(137) $$\gamma \left(R\right)=\bar{a}R^{4}+2\bar{b}R^{7}.$$

The other generator, β(R), is determined by $\bar{D_{s}}\left(c\right)$, its first and second derivatives evaluated at c=1, and ∂γR/∂R to be:

(138) $$\beta \left(R\right)=\frac{2\bar{b}R^{7}-{\left(\bar{a}R^{4}+2\bar{b}R^{7}\right)}^{2}}{4\bar{a}R^{3}+14\bar{b}R^{6}}\approx \left(\frac{\bar{b}R^{7}}{4\bar{a}R^{3}}\right).$$

The final approximation follows from $\bar{a}\gg \bar{b}$ after expanding the denominator in powers of $\bar{b}/\bar{a}$, applying a geometric series expansion, and only retaining terms of order $O(R^{6})$ and lower. Altenberger and Dahler now offer the approximation that the dependence of $\partial \bar{R}\left(c,R\right)/\partial c$ on $\bar{R}$ for c≠1 is given by the dependence of β(R) on *R* at c=1 by replacing *R* in the latter with $\bar{R}$. With this approximation:

(139) $$\frac{\partial \bar{R}}{\partial \ln c}=\frac{\bar{b}\;{\bar{R}}^{6}}{2\bar{a}}.$$

Noting $\bar{R}\left(c\right){|}_{c=1}=1$, an integral with respect to ln(c) yields:

(140) $$\bar{R}\left(c\right)={\left[1-\frac{3}{2}\frac{\bar{b}}{\bar{a}}\ln\left(c\right)\right]}^{-1/3}.$$

$\bar{b}$ and $\bar{a}$ have opposite signs, with $\bar{b}/\bar{a}\ll 1$, and c≥1, so $\bar{R}\left(c\right)$ is well behaved. The prediction for $D_{s}\left(c\right)$ is:

(141) $$D_{s}\left(c\right)=D_{s}\left(1\right)\exp\left(\int_{1}^{c}dx\;\left(\bar{a}{\bar{R}}^{4}\left(x\right)+2\bar{b}{\bar{R}}^{7}\left(x\right)\right)\right).$$

with the functional behavior of $\bar{R}\left(x\right)$ appearing in Equation (140). Ref. [41] performed a numerical integration of these equations, showing that $D_{s}\left(c\right)$ is very nearly a simple exponential in *c*, and that the calculated $D_{s}\left(c\right)$ is very nearly independent of $c_{r}$ so long as $c_{r}$ is small enough that $D_{s}\left(c_{r}\right)\approx 1$. The reference further noted evidence from the viscosity measurements that the renormalization group development could have an interesting fixed-point structure, but that here only the fixed point at the origin would be taken into account.

As the final step in the analysis, the issue of the concentration dependence of $R_{g}$ was considered at the level of approximation of the Daoud formula, in Equation (115). The proposed approach to calculating $D_{s}\left(c\right)$ was to imagine using the positive-function renormalization group separately for each final concentration, in each case performing the process with chains whose size was independent of concentration but which were the correct size for the target final concentration. The needed integration of Equation (141) was analytically performed by limiting terms to the $O(R^{4})$ level, leading to:

(142) $$D_{s}=D_{o}\exp\left(-\bar{a}R_{g}^{4}c^{1}\right)$$

or finally:

(143) $$D_{s}=D_{o}\exp\left(-\bar{a}R_{o}^{4}c^{1-2x}\right).$$

which is the universal scaling equation. The above analysis finds that this result is the $O(R^{4})$ approximation of a more accurate result.

Reference [41] also demonstrates that exponentials and stretched exponentials in *c* and *R* are invariants of the positive-function group transformation: If you start with a stretched exponential in *c* and *R*, you end up with a stretched exponential in *c* and *R* as the outcome of the renormalization transformation.

## 8. Polymer Solution Viscoelasticity from Two-Parameter Temporal Scaling

We now make a change of pace. The use of renormalization group procedures to extrapolate $D_{s}\left(c\right)$ and η(c) to elevated concentrations suggested using renormalization group approaches to infer the frequency dependencies of those properties. In the above, the calculations of hydrodynamic interactions were primary, with the self-similarity or the positive-function renormalization group being used to extend those calculations to elevated polymer concentrations. In this section, we focus almost entirely on the renormalization group properties of the calculation, deducing the aspects of the fixed-point structure of the renormalization group for the viscosity from empirical evidence. We then extend this analysis to a two-parameter form, thereby inferring the functional form for the frequency dependence of the loss and storage moduli.

The approach was put into effect in Ref. [42], which introduced a two-parameter temporal scaling to calculate how the loss modulus $G^{\prime \prime }\left(\omega \right)$ depends on frequency. The approach was entirely successful so far as it went, but has limitations that still need to be overcome. First, temporal scaling predicts the functional dependence of $G^{\prime \prime }\left(\omega \right)$ and therefore the storage modulus $G^{\prime }\left(\omega \right)$ on ω; however, in its current form temporal scaling gives no information on any numerical parameters found in the predicted functions. Temporal scaling does not yet predict how those parameters depend on polymer concentration or molecular weight, let alone what values the parameters have. Second, temporal scaling does not invoke a molecular model of a polymer solution. As a result, its predictions are substantially noncommunicating with the treatments of polymer viscoelasticity that begin with detailed models for molecular motions and intermolecular forces, such as those by Graessley [87,88], Bird et al. [89,90], and Raspaud et al. [91].

### Two-Parameter Temporal Scaling: Fundamental Approaches

The two-parameter temporal scaling approach has five theoretical parts and an experimental confirmation.

The five theoretical parts lead us to the frequency dependence of $g^{\prime \prime }\left(\omega \right)$. First, the renormalization group derivation of the universal scaling equation for $D_{s}$ is used to treat the low-shear solution viscosity η. Second, the phenomenological [21] behavior of the solution viscosity is examined. Third, the experimental phenomenology for η is used to infer the fixed-point structure of the full renormalization group treatment of η(c). Fourth, we advance from one- to two-parameter scaling by recognizing that η(c) is the low-frequency limit of η(c,ω). Fifth, from the inferred fixed-point structure of the associated renormalization group, we infer how η(c,ω) depends on ω at a fixed *c*. Finally, a comparison is made with the experimental literature, finding that the two-parameter temporal scaling approach correctly predicts the observed frequency dependencies. In more detail:

First, as discussed above, the hydrodynamic scaling model for self-diffusion leads to power series for $D_{s}$, which the positive-function renormalization group approach transforms into an exponential concentration dependence for $D_{s}$. The corresponding hydrodynamic calculation for the viscosity, and the same renormalization group approach, leads to an exponential concentration dependence for η. In each case, the effect of chain contraction with increasing polymer concentration is to replace the simple-exponential concentration dependence with a stretched-exponential concentration dependence.

The remainder of the analysis only invokes the renormalization group aspect of the calculation, and depends not at all on the assumption of the hydrodynamic scaling model that interchain interactions in the solution are dominated by hydrodynamics. If interchain interactions were instead dominated by chain crossing constraints or by *cryptocrystallites* [92], the low-concentration behavior was still a power series in concentration, the renormalization group part of the analysis would only suffer quantitative changes.

Second, there is an extensive experimental phenomenology for polymer solution viscosity. Reviews [21,59] of nearly the entirety of the phenomenological literature on η(c) find that η(c) indeed has the predicted stretched-exponential concentration dependence. In many but not all systems, there is an elevated concentration $c^{+}$ above which η instead depends on *c* as a power law:

(144) $$\eta =\bar{\eta }c^{x}$$

in *c* and not as a stretched exponential in *c*. Here, $\bar{\eta }$ and *x* are phenomenological constants. We describe the transition at $c^{+}$ as the *solutionlike-meltlike transition*. When the transition occurs, the transition concentration is typically $c^{+}\left[\eta \right]\approx 24-40$, with [η] being the intrinsic viscosity. In other systems, $c^{+}\left[\eta \right]$ is found to be as large as 150 or as small as 4. In yet other systems, no transition is observed. In all systems, the stretched-exponential curve admits Equation (144) as a local tangent. This local tangential behavior is not the solution-like–melt-like transition.

Milas et al. [93] and Graessley et al. [94] report viscoelastic parameters in various studies, including the low-shear viscosity, steady-state compliance $J_{e}^{o}$, and characteristic shear rate ${\dot{\gamma }}_{r}$ for non-Newtonian behavior for the solutions of linear [93,94] and star polymers [94]. These results were systematically reanalyzed [37]. For each system examined, all viscoelastic parameters measured consistently showed either the same solution-like behavior or the same melt-like behavior. When a solution-like–melt-like transition occurred, it occurred at the same concentration for all parameters measured.

Third, the transition at $c^{+}$ might be envisioned to have either a physical or a mathematical basis. As a physical transition, at $c^{+}$, there could be a crossover in the nature of the dominant force controlling the solution dynamics. For example, the crossover could be from domination by hydrodynamic interactions at lower concentrations to domination by chain crossing/entanglement interactions at elevated concentrations. On the other hand, as a mathematical transition, there could be a change in the nature of the mathematical solutions, for example, because the identity of the fixed point controlling the renormalization process changes with increasing *c*.

A physical transition might plausibly occur at the same c[η] in different systems (because chain crossing constraints are not sensitive to the chemical details of the chain structure), covering a wide range of concentration (because near $c^{+}$, the dominant forces would be competitive), and show a discontinuity in dη/dc near $c^{+}$ (because there is no reason for the different forces that dominate below and above $c^{+}$ to give the same slope as $c\to c^{+}$). On the other hand, a mathematical transition might plausibly occur over a narrow range of concentrations, occur at very different c[η] in different systems, and be analytic (first derivative dη/dc continuous) at $c^{+}$.

As it happens, the transition in the concentration dependence of η shows precisely the traits expected for a mathematical transition. Furthermore, a transition that is rather similar to the solution-like–melt-like transition is seen for η(c) of spherical microgel melts and hard-sphere colloids, so the transition cannot be due to any hypothetical crossover to chain reptation at elevated polymer concentrations. After all, spheres cannot reptate. *For the purpose of motivating the investigations of the remainder of this section, we take as a postulate that $c^{+}$ marks a mathematical fixed-point transition.* The low-concentration stretched-exponential behavior corresponds to the fixed point at c=0, while the elevated-concentration power-law behavior corresponds to a fixed point at some large concentration.

Fourth, the discussion thus far has taken η to be a function of the single variable *c*. However, polymer solutions are viscoelastic. Their viscoelastic responses are characterized by a frequency-dependent loss modulus $G^{\prime \prime }\left(\omega \right)$ and a frequency-dependent storage modulus $G^{\prime }\left(\omega \right)$. The moduli are also concentration-dependent, but in the usual convention, one writes $G^{\prime \prime }\left(\omega \right)$ and not $G^{\prime \prime }\left(\omega ,c\right)$. The viscosity η(c) is the low-frequency limit of a frequency-dependent viscosity:

(145) $$\eta \left(c,\omega \right)=\frac{G^{\prime \prime }\left(\omega \right)}{\omega }.$$

The discussion so far considers η(c), so when we extend to frequency dependence, we consider $G^{\prime \prime }\left(\omega \right)/\omega$ and $G^{\prime }\left(\omega \right)/\omega^{2}$ and not $G^{\prime \prime }\left(\omega \right)$ or $G^{\prime }\left(\omega \right)$. $G^{\prime \prime }\left(\omega \right)/\omega$ and $G^{\prime }\left(\omega \right)/\omega^{2}$ have the appropriate property that they go to constants when ω→0.

Fifth, it is assumed that η(c,ω) is dominated by the same fixed points that determine the behavior of ${\eta \left(c,\omega \right)|}_{\omega \to 0}$. Consider a (c,ω) plane, the *c* axis being horizontal and the ω axis being vertical. If one proceeds away from (c,ω)=(0,0) by moving along the line ω=0, one observes the dependence of η(c,0) on *c*. With increasing *c*, one eventually encounters the solution-like–melt-like transition. At smaller *c*, η(c,0) depends on *c* as a stretched exponential, corresponding to a renormalization group fixed point at the origin. Above the transition, η(c,0) depends on *c* as a power law in *c*, corresponding to the dominance of a second fixed point that is not at the origin. If, instead of staying at ω=0, one instead advanced away from the ω=0 axis by moving perpendicular to the ω=0 axis, thereby staying at fixed *c*, the same fixed points would control the behavior of η(c,ω). The ω dependence would then be a stretched exponential at smaller ω, due to the fixed point at (0,0), and a power law at larger ω, due to the second fixed point at some larger (c,ω).

(146) $$\frac{G^{\prime \prime }\left(\omega \right)}{\omega }=\left\{\begin{array}{cc} G_{20}\exp\left(-\alpha \omega^{\delta }\right), & \mathrm{if}\;\omega \leq \omega_{t}\\ {\bar{G}}_{20}\omega^{-x}, & \mathrm{if}\;\omega \geq \omega_{t}. \end{array}\right.$$

Similar two-case formulae are expected to describe $G^{\prime }\left(\omega \right)/\omega^{2}$ and η(κ), where κ is the shear rate. The six parameters $G_{20}$, α, δ, $\omega_{t}$, $\bar{G_{20}}$, and $\omega_{t}$ are numerical constants appropriate to the particular polymer, its molecular weight, and its solution concentration. Because the transition is predicted and found to be continuous and analytic (functions and first derivatives the same at the transition frequency $\omega_{t}$), these six parameters are not all independent from each other. Between them, there are only four independent parameters.

The ansatz given here is not a complete derivation. However, as shown in the original paper [42] and in Ref. [21], chapter 13, Equation (146) and the corresponding equations for $G^{\prime }\left(\omega \right)/\omega^{2}$, and separately for η(κ), are in excellent agreement with the experimental studies of the storage and loss moduli and of shear thinning. Furthermore, as would be expected for physically significant variables, the six parameters found in these equations all show smooth dependencies, often power laws, on *c*.

## 9. Brief Description of Individual Historical Papers

In this section, we present the short summaries of the papers that developed and tested the hydrodynamic scaling model. The original paper in the series was Ref. [22], Phenomenological Scaling Laws for “Semidilute” Macromolecule Solutions from Light Scattering by Optical Probe Particles, which was the first systematic review of optical probe particles diffusing through matrix polymer solutions. The probes were polystyrene latex spheres and bovine serum albumin, diffusing through solutions of several water-soluble polymers and bovine serum albumin. Probe motion was determined using quasi-elastic light scattering. The paper set themes for later work, identified areas that were explored later, and made it clear that the experiment did not match some contemporary models. The probe diffusion coefficient $D_{p}$ was found to follow the stretched exponential:

(147) $$D_{p}=D_{p0}\exp\left(-\alpha c^{\nu }M^{\gamma }R^{\delta }\right),$$

with *c*, *M*, and *R* being the polymer concentration and molecular weight, and the probe radius, respectively, $D_{p0}$ and α being constants. The exponents were found to be ν=0.6−1.0, γ=0.8±0.1, and δ ranging from 0 to −0.1, contrary to some theoretical expectations [95] that one should find γ=0 and δ=1. For small polymers, $D_{s}$ tracked the solution fluidity $\eta^{-1}$. For large (M≥100 kDa) polymers, probes diffused faster than expected from $\eta^{-1}$, even when the probes were extremely large (R≈0.62μm).

The successor paper [23] Universal Scaling Equation for Self-Diffusion by Macromolecules in Solution extended the work in the previous paper to the polymer self-diffusion coefficient $D_{s}$. It was shown that the then-available measurements of $D_{s}$ at elevated concentration uniformly fit stretched exponentials in *c*, but did not fit the power laws predicted by some scaling models. Also, the stretched exponential accurately described $D_{s}\left(c\right)$ over a full range of concentrations, with no indication of a discontinuity at some elevated ’entanglement’ concentration $c^{*}$. These results were not widely expected on the basis of other polymer models, leading to the criticism that the finding was purely phenomenological, and the emphatic suggestion [68] that a derivation of the universal scaling equation was needed.

The skeleton of a derivation for the universal scaling equation for polymer self-diffusion was soon supplied [23]. Dynamics of Polymers in Concentrated Solution: The Universal Scaling Equation Derived obtained the stretched exponential for $D_{s}$. The model was non-reptational; collective (hydrodynamic) modes were taken to dominate the local (entanglement) mode at all concentrations. The derivation reached the stretched exponential via a self-similarity argument, an assumed form for chain–chain hydrodynamic interactions, and the known tendency of random-coil polymer coils to contract in concentrated polymer solutions. The model predicted that $\alpha ∼M^{1}$, and that ν changes from 1 to 1/2 with increasing polymer molecular weight—results which were confirmed by the review of the literature.

The mathematical structure of the derivation did not rely on transport properties unique to self-diffusion. The same approach, with numerical modifications reflecting the quantity being calculated, was therefore expected to be applicable to other transport properties. Indeed, this author and Peczak [25] showed in The Ubiquity of Stretched-Exponential Forms in Polymer Dynamics that the polymer solution transport properties generally follow stretched-exponential concentration dependencies.

A simplest form of Kirkwood–Riseman model [27] for polymer dynamics appears in Quantitative Prediction of α in the Scaling Law for Self-Diffusion, where it was used to compute α. For 1 MDa polystyrene, the calculation gave α=2, while α≈0.7 is the experimental number. As *M* is reduced, the calculated α decreases more rapidly than the measured α, so the error in the calculated α at smaller *M* is closer to 50% than it is to a factor of three. However, the calculation did not incorporate intrachain hydrodynamics, and took the distance of the closest approach of two polymer chains to be twice the monomer radius, both of these approximations tending to increase α, so it is not surprising that our approximation for α gave a value larger than the experimental one.

The calculation of Ref. [27] was extended to treat self-diffusion by star polymers. In Chain Architecture in the Hydrodynamic Scaling Model, it was shown [29] that if one compares the self-diffusion of linear polymers and of many-armed star polymers, the polymers being of equal total molecular weight, a solution of matrix polymers is modestly more effective at delaying the linear polymer. However, if one compares the self-diffusion of the linear and star polymers at an equal arm molecular weight, a linear polymer being a two-armed star, the matrix polymer is far more effective at delaying the star polymer than at delaying the linear polymer.

The short paper The Hydrodynamic Scaling Model for Polymer Dynamics [30] notes a series of experimental tests distinguishing between the hydrodynamic scaling and reptation-scaling models, including (i) the presence or absence of multiple dynamic regimes, (ii) the difference or lack of difference between sphere and random-coil polymer diffusion, and (iii) power-law or stretched-exponential concentration and molecular weight dependencies of $D_{s}$ and η, the strong or weak effect of probe radius on $D_{p}/D_{p0}$, and the effect of chain architecture on $D_{s}$. For every test, hydrodynamic scaling is preferred to reptation-scaling, showing that the ongoing theoretical project to refine the hydrodynamic scaling model was on the right track. The paper echoed the analysis in the extended article The Hydrodynamic Scaling Model for Polymer Self-Diffusion [28], particularly that there was a need for substantial additional measurements on the solutions of small-*M* (say, ≤100 kDa) polymers and a requirement that measurements should be systematically carried down to a zero matrix concentration. Furthermore, based on particular studies of how $D_{p}$ depends on *R*, it was clear that polymer solutions are qualitatively not like chemically cross-linked gels, even on short time scales.

The paper Range of Validity of the Hydrodynamic Scaling Model [33] observes that solvent-mediated interactions are absent in the melt (except as one views the melt as its own solvent), and therefore with increasing concentration, there should be a transition in dynamic behavior. In this paper, the transition was identified with the change from the stretched-exponential to the power-law concentration dependencies. This interpretation was not sustained by more modern work, but the solution-like–melt-like transition still played an important role in understanding the polymer dynamics.

In their paper Higher-Order Hydrodynamic Interactions in the Calculation of Polymer Transport Properties, Phillies and Kirkitelos [35] examined the consequences of higher-order bead–bead interactions. Bead–bead interactions are usually modeled using the Oseen tensor. However, it is entirely clear from the calculations of the self- and mutual-diffusion coefficients of colloidal spheres [96] that the Oseen tensor is totally inadequate as an approximation for the true hydrodynamic interaction tensor, and that higher-order (in a/R, *a* being the bead radius and *R* being the distance between beads) terms must be included. Phillies and Kirkitelos included higher-order terms in calculating $D_{s}$. Furthermore, they calculated the effect of interchain interactions on the polymer bead and free-monomer mobilities, showing that the effects are not the same. Inferring a concentration dependence for the friction coefficient of individual polymer beads from the concentration dependence of the friction coefficient of free small molecules in solution (the monomer friction coefficient correction) is therefore fundamentally invalid.

Phillies and Quinlan [36], in Analytic Structure of the Solutionlike-Meltlike Transition in Polymer Solution Dynamics, reported a high-precision detailed study of the analytic structure of the solution-like–melt-like transition in the viscosity. η(c) of several hydroxypropylcellulose samples shows a stretched-exponential concentration dependence at smaller *c* and a power-law concentration dependence at larger *c*. Phillies and Quinlan showed that the viscosity transition is analytic—the functions and their first derivatives are both continuous—at the transition concentration. A later analysis of the literature in Viscosity of Hard Sphere Suspensions [46] shows that hard and soft-sphere suspensions show the same solution-like–melt-like transition in η(c), at a concentration well below the concentration of the known phase transition, thus demonstrating that the solution-like–melt-like transition does not arise from the topological effects unique to long linear polymers.

Writing in Hydrodynamic Scaling of Viscosity and Viscoelasticity of Polymer Solutions, Including Chain Architecture and Solvent Quality Effects, Phillies [37] applied the universal scaling equation and power law forms to the concentration and molecular weight dependencies of various viscoelastic parameters, including the results on linear and star polymers and systems having various solvent qualities. This paper was primarily a phenomenological study; model calculations corresponding to the viscoelastic parameters have not yet been made.

The paper Quantitative Experimental Confirmation of the Chain Contraction Assumption of the Hydrodynamic Scaling Model [39] takes advantage of a unique feature of dielectric relaxation spectroscopy, namely with some polymers, the technique can measure both the rotation of the chain end-to-end vector as well as the length of that vector. The hydrodynamic scaling model asserts that the deviation of the concentration dependence of transport properties from a simple exponential in concentration is caused by chain contraction at elevated polymer concentrations. The dielectric relaxation measurements confirm that the model’s assertion is quantitatively exact.

Phillies et al.’s [40] paper Probe Diffusion in Sodium Polystyrene Sulfonate—Water: Experimental Determination of Sphere-Chain Binary Hydrodynamic Interactions made a quantitative test of the hydrodynamic model used here. The $D_{c}$ of three sizes of polystyrene sphere was determined in the solutions of seven different monodisperse polystyrene sulphonates (1.5≤M≤1188 kDa), each at ten or more concentrations of up to 20 g/L. The initial slopes $dD_{p}{/dc|}_{c\to 0}$ were compared with theory. The one uncertainty is the diameter to be assigned to the polymer’s monomeric subunits. Fortunately, for large probes, the slopes are very nearly independent of the assumed subunit diameter. For M>10 kDa, quantitative agreement between the measurement and theory was obtained, confirming the validity of the hydrodynamic calculation.

The paper Derivation of the Universal Scaling Equation of the Hydrodynamic Scaling Model via Renormalization Group Analysis [41] replaced the self-similarity approach of Ref. [23] with a calculation based on the positive-function renormalization group [49,50]. The hydrodynamic scaling model’s universal scaling equation was again obtained, via a very different approach.

The use of renormalization group techniques in the prior paper led to a much more radical paper [42] Polymer Solution Viscoelasticity from Two-Parameter Temporal Scaling, which proposed to find the frequency dependence of the loss modulus from a consideration of the fixed points of a hypothetical renormalization group derivation of the viscosity η(c), made on the lines of Ref. [41], after identifying η(c) as a one-dimensional slice of η(c,ω). The ansatz in the paper leads to the conclusion that $G^{\prime \prime }\left(\omega \right)/\omega$ is a stretched exponential in ω at lower frequencies and a power law in ω at elevated frequencies, the transition between the two regimes being continuous and analytic. Preliminary tests against the literature data were highly satisfactory. Further tests reported in Temporal Scaling Analysis: Viscoelastic Properties of Star Polymers [43], Temporal Scaling Analysis: Linear and Crosslinked Polymers [44], and Viscosity of Hard Sphere Suspensions [46] were equally satisfactory. In particular, it was confirmed that the predicted forms, with fitted parameters, agreed with Kronig–Kramers relations. Furthermore, the forms that effectively describe the $G^{\prime \prime }\left(\omega \right)$ and $G^{\prime }\left(\omega \right)$ of linear polymers also effectively describe $G^{\prime \prime }\left(\omega \right)$ and $G^{\prime }\left(\omega \right)$ of spherical microgel melts, showing that the chain topology does not make a qualitative contribution to the functional forms of the loss and storage moduli.

In Low-Shear Viscosity of Non-Dilute Polymer Solutions from a Generalized Kirkwood-Riseman Model [45], the model of Ref. [27] was employed to calculate the concentration dependence of the viscosity, including interacting pairs and the trios of polymer chains. An extended computation leads to values for the initial slope dη/dc and for the Huggins coefficient. The results of this calculation were used in Self-Consistency of Hydrodynamic Models for the Low-Shear Viscosity and the Self-Diffusion Coefficient [6] to calculate α of Equation (5). Taking the Huggins coefficient from the viscosity calculation as an experimental input, α for self-diffusion was determined, as a function of the polymer molecular weight, with no free parameters. The comparison with experimental determinations α found almost exact agreement over four orders of magnitude in *M* and α.

Finally, the renormalization group treatment of $D_{s}\left(c\right)$ requires as input a power-series expansion for $D_{s}\left(c\right)$. As the first step toward advancing on that expansion, in Fourth-Order Hydrodynamic Contribution to the Polymer Self-Diffusion Coefficient [47], Merriam and Phillies used a hydrodynamic multiple-scattering approach to compute the chain–chain–chain–chain–chain hydrodynamic interaction tensor, which could be used to calculate the $c^{3}$ correction to $D_{s}$.

## 10. Phenomenological Evidence

This section presents phenomenological evidence on polymer dynamics. As will be seen, the evidence supports the hydrodynamic scaling model. In understanding the evidence and what it means, it is worthwhile to begin with the philosophical observations of Thomas Kuhn [97] on how theories compete. Kuhn’s fundamental thesis is that one’s model of the world influences which experiments need to be made, which quantities need to be calculated or elsewise predicted, and which sorts of data are important. An experiment that is viewed as a critical test of one model may for a different model appear to only be of marginal relevance. In the end, a successful model predicts all experimental observations within its scope, but at the earlier stages of adoption, some sorts of measurements taken to be central and others are taken to be marginal, to be considered later.

As an example of the above matter, Kuhn treated the early-19th century competition between phlogiston models for chemical structure and Dalton’s law of multiple proportions. The phlogiston model was widely accepted because it was extremely successful. It ordered and explained vast amounts of descriptive chemical information, for example, the model explained why pure metals are more similar to each other than their oxides are similar to each other. While it was entirely possible to weigh the amount of each element needed to form a particular chemical compound, within the phlogiston picture, such measurements did not appear to matter. Dalton’s law of multiple proportions put that interpretation on its head, proposing that the weight of each element in a pure compound was the central chemical fact. The descriptive material explained well by the phlogiston models, such as the colors of the metallic oxides, were set aside, to be explained as it turned out a century and a half later with quantum mechanics. Materials that were in fact the solid solutions of several compounds, leading to material substances in which the law of multiple proportions was not followed, were viewed as anomalous special cases—not as disproofs of the model. In moving from the phlogiston model to Dalton’s law, not only did science change how one described matter, but it also changed which experimental findings were to be treated as marginal results, and which experimental findings were to be treated as central tests of the theory.

The difference in world view between the hydrodynamic scaling and reptation-scaling models already arises in data presentation and experimental plans. The models predict that transport coefficients depend on concentration and molecular weight as stretched exponentials and as power laws, respectively. Furthermore, the hydrodynamic scaling model, if correct, is valid from dilute solution up to some large concentrations, while reptation-scaling models, if correct, are only valid at concentrations above some overlap concentration $c^{*}$ and extending to the melt. In consequence, an experiment whose plan arises from hydrodynamic scaling concepts includes measurements on dilute as well as concentrated solution behavior. Experiments whose plans arise from reptation-scaling concepts often only report measurements on solutions having $c>c^{*}$, and are therefore not always helpful for testing hydrodynamic scaling. Correspondingly, in making the graphical presentations of transport coefficients against *c* or *M*, data testing reptation-scaling models are usefully set out on log-log plots, while data arising from hydrodynamic scaling models are usefully set out on linear or semilog plots.

The following sections treat the experimental tests of various aspects of the hydrodynamic scaling model. Section 10.1 presents an experimental test of the accuracy of the generalized Kirkwood–Riseman model for intermacromolecular hydrodynamic interactions. Section 10.2 considers the tests of the predicted functional dependencies of $D_{p}$ on *c* and *M*. Predictions for $D_{s}$ and η require a notional bead radius *a*. Section 10.3 demonstrates that the values of *a* from $D_{s}$ and from η are mutually consistent. In the model, non-exponentiality arises from chain contraction; Section 10.4 uses the literature on the dielectric relaxation of polymers in the solution to demonstrate that chain contraction quantitatively accounts for the deviations of the concentration dependence of the dielectric relaxation time from simple exponential behavior. Chain contraction and expansion are also affected by solvent quality. Section 10.5 examines the results of Dreval et al. [98] that compare concentrated-solution viscosity and solvent quality, and the results of Phillies and Clomenil [34] on probe diffusion through polymers in good and theta solvents. Finally, at some concentration, the simple model presented here is obliged to become inapplicable, because the polymer molecules are too close together to treat the solvent as a continuum. When this concentration is exceeded, a transition must take place. Section 10.6 presents evidence that this predicted transition has been observed.

### 10.1. Measurements of the Hydrodynamic Interaction Tensor

The simplest test of the Kirkwood–Riseman model refers to a single polymer chain. In the Kirkwood–Riseman model, the self-diffusion coefficient of a single random-coil chain is substantially determined by hydrodynamic interactions between the different parts of the same chain. A test of the Kirkwood–Riseman model for isolated chains is reported by Pesce et al. [99] studying intrinsically disordered proteins and regions. They made molecular dynamics simulations on a series of 11 such proteins. For each protein, they calculated the average size and the self-diffusion coefficient as predicted by the Kirkwood Riseman model. Comparison with small-angle X-ray scattering confirmed that their size calculations were accurate. Comparison with pulsed-gradient spin-echo nuclear magnetic resonance measurements confirmed that the Kirkwood–Riseman model accurately predicts the self-diffusion coefficient, i.e., the Kirkwood–Riseman model accurately treats the hydrodynamics of polymer random coils.

The hydrodynamic scaling treatment is based on an extended Kirkwood–Riseman model. Is the extended Kirkwood–Riseman model accurate? Phillies, Lacroix, and Yambert [40] made a quantitative experimental test of the model of Section 3. The test was successful. They used quasi-elastic light scattering spectroscopy to measure the diffusion coefficient $D_{p}$ of polystyrene latex spheres of three known sizes through the solutions of seven polystyrene sulphonates, 1.5≤M≤1190 kDa, at a series of polymer concentrations. Anomalous polyelectrolyte effects were suppressed by working in 0.2M NaCl. The initial slopes $\alpha =\lim_{c\to 0}dD_{p}/dc$ were determined.

Comparison was then made with the hydrodynamic calculations of Phillies and Kirkitelos [35], in which bead–bead hydrodynamic interactions were truncated at the $r^{-3}$ (Rotne–Prager) level. Probe spheres were treated as a single polymer bead having a known large radius. What size *a* should be assigned to the monomer beads in the polymer? The algebraic answer is a function of the sum of the monomer and probe radii. The polymer beads were much larger than the polymeric monomer units, so the exact size assigned to the monomers has little effect on the calculation. The polymer radii of gyration and hydrodynamic radii were calculated from their molecular weights using the data of Pietzsch et al. [100]. The Phillies–Kirkitelos calculation thus *has no free parameters*. It predicts numerical values of α. For M<10 kDa, it appears inaccurate to model the somewhat rigid polystyrene sulphonate as a Gaussian-random cloud of monomer beads. For polymer M>10 kDa, nearly quantitative agreement between the calculated and measured values of α was found, as seen in Ref. [40], Figure 4. α for a 1 MDa polymer was calculated to be ≈0.15, and was experimentally found to be ≈0.13. Over nearly a hundred-fold range of polymer molecular weights, $\alpha ∼M^{\gamma }$ for γ=1 was found, in agreement with the Phillies and Kirkitelos calculation.

These experimental results directly confirm the validity of the hydrodynamic approach for calculating interchain hydrodynamic interactions, at least for the self terms $b_{ii}$ of the calculated mobility. These experiments did not test self-similarity, the positive-function renormalization group process, or the size of the two-chain tensor $T_{ij}$.

### 10.2. Concentration and Molecular Weight Dependencies

The hydrodynamic scaling model, via either self-similarity or the positive-function renormalization group, predicts that transport coefficients have stretched-exponential dependencies on the concentration and molecular weight. The scaling prefactor α and scaling exponent ν of the stretched exponential are predicted by the model to depend on *M* but to be independent of polymer concentration. The PFRG mathematical structure has a route permitting a transition to a power-law concentration dependence at large *c*.

Experimentally [101], $D_{s}$, $D_{p}$, and η follow stretched exponentials in *c*, beginning at extreme dilution and extending out to an elevated polymer concentrations, often c[η]≫1. For $D_{s}$ and $D_{p}$, there are no indications of a discontinuity or change in slope of the concentration dependence for some c[η] near unity. The lack of a discontinuity agrees with the hydrodynamic scaling model presumption that the same dynamics apply in dilute and non-dilute solutions. The same lack of a discontinuity rejects proposals that polymer dynamics change qualitatively, in a way that affects the concentration dependence, at some concentration $c^{*}\approx {\left[\eta \right]}^{-1}$, at which polymer chains overlap and entangle.

The hydrodynamic scaling model for $D_{p}$ predicts that $\alpha ∼M^{\gamma }$ for γ≈1; also, with increasing *M*, ν should decrease from 1 to 5/8 or 1/2. Phillies and Quinlan measured η and $D_{p}$ of 20.4 and 230 nm polystyrene spheres, for dextran solutions having *M* in the range of 10–500 kDa and a range of concentrations. Values for α and ν were extracted from each set of measurements. Over nearly two orders of magnitude in *M*, $\alpha ∼M^{0.84}$, while as predicted by the model, ν decreased from 1 to 5/8.

If the matrix polymer is replaced with a globular matrix species such as a protein, $D_{p}\left(c\right)$ for probe spheres diffusing through the protein solution continues to have a stretched-exponential form. This result is consistent with the hydrodynamic scaling expectation that replacing a random-coil matrix with a hard-sphere matrix changes numerical coefficients in the hydrodynamic interaction tensor, but has no qualitative effect on $D_{p}\left(c\right)$. This result is inconsistent with reptation-tube model expectations that the dynamics of entangling and non-entangling matrix species should not be similar at high concentrations [66].

A comparison [29] of $D_{s}$ for linear and star polymers diffusing through a matrix solution of dissolved linear polymers, as studied by Wheeler and Lodge [102,103], finds that a large concentration of linear polymers is approximately equally effective at delaying the motion of linear and star polymers having the same total molecular weight. However, comparing linear and 12-armed star polymers having the same arm molecular weight, a linear polymer being a two-armed star, the same matrix solutions are far more effective at delaying the motion of the 12-armed star than at retarding the motion of a linear polymer. These measurements of Wheeler and Lodge [102,103] were shown by this author [29] to be consistent with the hydrodynamic scaling model.

At elevated polymer concentrations, $D_{p}$ often shows non-Stokes–Einsteinian behavior, i.e., $\kappa \equiv D_{p}\left(c\right)\eta \left(c\right)/D_{p}\left(0\right)\eta \left(0\right)\gg 1$, up to $\kappa \approx 10^{3}$, even for large (e.g., 1 μm diameter) probes. Non-Stokes–Einsteinian behavior is equally found for probes in non-entangling bovine serum albumin solutions [66,101], showing that non-Stokes–Einsteinian behavior is not an indicator for the presence of reptational motion by the matrix.

For η(c), ${\dot{\gamma }}_{r}$, and $J_{e}^{o}$, in some systems but not others, the concentration dependencies show at elevated *c* a transition from a stretched-exponential to a power-law concentration dependence [37]. On one hand, reptation-tube models do predict scaling (power law) behavior at a large *c*. On the other hand, reptation-tube models indicate that the transition should be universal, appreciably independent of the details of a polymer chemical structure, and therefore should consistently appear at about the same c[η]. Experimentally, the transition is not uniform and occurs at greatly different concentrations c[η] in different systems, contrary to expectations from tube-type models.

Tube-type models for concentrated solutions ascribe to polymer chains a mode of motion—reptation—that is inaccessible to large spheres, implying that chains will diffuse through the concentrated solutions of large polymer molecules considerably more rapidly than will spheres that have the same hydrodynamic radius. Brown and Zhou [104,105] compared the $D_{p}$ of spheres and $D_{s}$ of random coil probes through the solutions of the same polymer. For large probes and chains in the solutions of a smaller matrix polymer, $D_{p}\left(c\right)/D_{s}\left(c\right)$ was approximately independent of the concentration as $D_{p}\left(c\right)/D_{p}\left(0\right)$ declined by more than two orders of magnitude. For smaller probes in the solutions of a large matrix polymer, with increasing matrix *c*, the matrix polymer was much more effective at retarding the motions of the random coil polymer than at retarding the motion of spherical probes. Their findings are consistent with hydrodynamic scaling models that view hydrodynamic radii as the central variable, but are inconsistent with tube-type models.

### 10.3. The Bead Diameter a

Calculations of the concentration dependencies of the self-diffusion coefficient and the viscosity lead to outcomes determined in part by a notional bead diameter *a*. It could be proposed that, in each of these calculations, there is a free parameter, so quantitative comparisons between the data and the theoretical model are impossible. (For probe diffusion, this difficulty does not arise, because *a* has little effect on $dD_{p}/dc$, as discussed in Section 10.1.)

*a* can be estimated from the viscosity calculation. This *a* will be denoted $a_{\eta }$. Pearson [106] and Yamakawa [107] reported that $k_{H}$ is in the range of 0.3–0.6. Noting for non-draining spheres $\left[\eta \right]=2.5\bar{v}$ and in appropriate units $\bar{v}=4\pi R^{3}/3$, one finds from Equation (108) $a_{\eta }=0.18R$. The estimated $a_{\eta }$ does not strongly depend on the assumed $k_{H}$.

Second, in Ref. [41], the same hydrodynamic approach was applied to the self-diffusion coefficient $D_{s}$, obtaining [41]:

(148) $$\alpha =-\frac{9}{16}\frac{R_{h1}^{2}}{R_{g}a_{D}}\frac{4\pi R_{g}^{3}}{3}\frac{N_{A}}{M}.$$

Here, $R_{g}$ is the radius of gyration, $R_{h}$ is the hydrodynamic radius, $a_{D}$ is the notional bead size inferred from self-diffusion, $N_{A}$ is Avogadro’s number, and *M* is the polymer molecular weight. For [108], $1.27\times 10^{6}$ Da polystyrene in benzene [108], $R_{g}\approx 620$Å, $R_{h}\approx 380$Å, and from a systematic review [21] of the published literature α≈−0.6 with *c* in g/L at this molecular weight. Combining these findings, $a_{D}\approx 0.17R_{g}$.

The two paths to estimating *a* indicate that *a* is 0.18R or $0.17R_{g}$, i.e., $a_{\eta }\approx a_{D}$. Given the approximations needed to reach this point, the two estimates of *a* agree within experimental error and calculational imprecision. The determinations of *a* from two separate types of data after separate calculations [23,27,35,41] based on the hydrodynamic scaling model lead to about the same notional bead diameter. This outcome would be expected if the hydrodynamic scaling model supplied the legitimate physical treatment, but is unlikely if $a_{\eta }$ and $a_{D}$ were simply fitting parameters that had no physical meaning.

### 10.4. Dielectric Relaxation Spectroscopy

Dielectric spectroscopy is sensitive to the size and temporal behavior of polymer dipole moments. The understanding of polymer dipole moments may be traced back to Stockmayer [109], who identified three classes of polymer dipole moment and their relaxations, namely: (1) dipoles whose orientation is determined by the orientation of pendant side groups, and which therefore change direction on very short time scales; (2) dipoles whose direction is determined by the chain contour, and which are aligned in perpendicular to the chain contour, so that they are relaxed via local segmental motion; and (3) dipoles associated with the chain contour that point along the chain contour, so that the magnitude of the dipole moment is determined by the end-to-end-vector of the polymer chain, and which change direction on the slow time scales on which the polymer and its end-to-end vector rotate in space. Stockmayer classed polymers with type 3 dipoles as *type-A polymers*. Dielectric relaxation spectroscopy associated with solutions of type-A polymers has been reviewed by Adachi and Kotaka [110].

A type-A polymer can be viewed as a series of short segments, where each segment *i* has a dipole moment $d_{i}$ aligned in parallel to the segment. The time-dependent dipole moment M(t) of the polymer is the sum of the moments of the *N* segments:

(149) $$M\left(t\right)=\sum_{i=1}^{N}d_{i}\left(t\right).$$

For identical segments, $d_{i}\left(t\right)∼r_{i}\left(t\right)$, $r_{i}\left(t\right)$ are the segment end-to-end vector. The mean-square of the polymer end-to-end vector $R_{e}$ is therefore $\langle R_{e}^{2}\rangle ∼\langle M\left(t\right)\cdot M\left(t\right)\rangle$. The dipole relaxation function:

(150) $$\Phi \left(t\right)=\frac{\langle M(t)\cdot M(0)\rangle }{\langle M(0)\cdot M(0)\rangle }$$

describes the relaxation of the polymer end-to-end vector. Φ(t) is usually said to describe rotational diffusion. However, for a random-coil polymer as opposed to a rigid body, the length of the end-to-end vector and hence |M(t)| fluctuates in time, so Φ(t) must capture the relaxation of fluctuations in the magnitude as well as the direction of M(t).

With dielectric relaxation, one can measure both Φ(t) (and hence, the polymer rotational diffusion coefficient) and $\langle R_{e}^{2}\rangle$. As demonstrated by Adachi and Kotaka [110] and by Ref. [39], the end-to-end vectors of the pairs of polymer molecules are almost certainly very nearly uncorrelated in direction, so dielectric relaxation spectroscopy measures single-chain properties, even in concentrated solutions. Adachi et al. [111,112,113] exploited their demonstration by measuring the dielectric relaxation strength Δϵ and relaxation time $\tau_{n}$ of the *cis*-polyisoprenes of multiple molecular weights in good and theta solvents at concentrations of 0–500 g/L. $\tau_{n}$ increases by as much as several hundredfold over this range. In the same systems Δϵ decreased with increasing *c*, in some cases, by as much as 50%.

Phillies [39] reconsidered the experimental findings of Adachi et al. [111,112,113]. Simple phenomenological forms that quantitatively describe the concentration dependence of $\langle R_{e}^{2}\rangle$ were identified. The chain–chain hydrodynamic interaction tensor for rotation–rotation coupling was proposed, based on the comparable sphere–sphere coupling, to scale as $R^{6}/r^{6}$, with *R* as a chain radius and *r* as a distance between chains. The 1997 analysis proposed that the ensemble average over chain positions had an effective lower limit a∼R, so that the self term of the rotation–rotation coupling had a strength $∼R^{3}$. Invoking the self-similarity rationale, the renormalization group treatment, which was not developed at the time of this paper, Ref. [39] proposed:

(151) $$\tau_{n}\left(c\right)=\tau_{n0}\exp\left(\alpha c{\langle {\left(R_{e}\left(c\right)\right)}^{2}\rangle }^{3/2}\right).$$

The effect of the concentration-dependent chain contraction is then to determine the curvature of $\tau_{n}\left(c\right)$.

For dielectric relaxation, $\tau_{n}$ and $\langle {\left(R_{e}\left(c\right)\right)}^{2}\rangle$ have been measured directly [111,112,113]. A fit of Equation (151) to the experimental measurements has two free parameters, namely $\tau_{n0}$ and α. On a semilog plot, these parameters give an intercept and an initial slope. However, any deviation of $\tau_{n}\left(c\right)$ from pure exponential behavior is determined by the known quantity $\langle {\left(R_{e}\left(c\right)\right)}^{2}\rangle$.

There is quantitative agreement between Equation (151), in its determination of the dependence of $\tau_{n}\left(c\right)$ on $R_{e}$, and the experiment. In theta solvents, the polymer sizes are independent from polymer concentration, so $\tau_{n}\left(c\right)$ is predicted to be a simple exponential, precisely as found. In good solvents, $\langle {\left(R_{e}\left(c\right)\right)}^{2}\rangle$ decreases with increasing *c*, leading to a $\tau_{n}\left(c\right)$ that increases less rapidly than a pure exponential, also as observed. The degree of non-exponential behavior is determined by the amount of chain contraction, as quantitatively confirmed in Ref. [39], precisely as predicted by the hydrodynamic scaling model and Equation (151). The results in Ref. [39] may be seen as a significant advance over the self-similarity and renormalization group treatments in one key respect, namely since an assumed theoretical dependence of $R_{g}$ on *c* has been replaced with the experimental dependence, thereby creating a quantitative agreement between the calculation and the experiment.

### 10.5. Viscosity and Solvent Quality

Dreval et al. [98] reviewed an extremely extensive set of viscoelastic studies not readily available in the Western literature. They considered a reduced viscosity $\tilde{\eta }=\left(\eta -\eta_{0}\right)/\left(\eta_{0}c\left[\eta \right]\right)$. For a series of homologous polymers in the same solvent, c[η] was found to be a good reducing variable, the intrinsic viscosity [η] collapsing η(c) for different *M* onto a single curve. However, when the same polymer samples were dissolved in several different solvents, the plots of $\tilde{\eta }$ against c[η] were found to lie on different curves. Dreval et al. showed that the various curves could all be reduced onto each other by introducing a new variable $K_{M}$ and plotting $\tilde{\eta }$ against $K_{M}c\left[\eta \right]$. With a correct choice of $K_{M}$ for each solvent:polymer pair, all measurements of $\tilde{c}$ of the same polymer in different solvents could be reduced to a single master curve.

Dreval et al. then introduced a chain expansion parameter $\alpha_{\eta }$, defined via:

(152) $$\alpha_{\eta }^{3}=\frac{{\left[\eta \right]}_{s}}{{\left[\eta \right]}_{\left[theta\right]}}.$$

Here, ${\left[\eta \right]}_{s}$ is the intrinsic viscosity of the polymer in the solvent of interest, and ${\left[\eta \right]}_{\left[theta\right]}$ is the intrinsic viscosity of the same polymer in a theta solvent, where it is unexpanded. Dreval et al. then demonstrated that $K_{M}\alpha_{\eta }^{3}$ is approximately a constant, i.e., $K_{M}∼\alpha_{\eta }^{-3}$. The solvent quality thus enters η(c) exactly as predicted by the hydrodynamic scaling model and seen in Equation (151), namely that η(c) is a function of $c{\langle {\left(R_{e}\left(c\right)\right)}^{2}\rangle }^{3/2}$, and $R_{e}$ is determined in part by the solvent quality. The reducing variable $K_{M}$ divides out the effect of the solvent quality on $R_{e}$, so that plots for a given polymer of η(c) against *c* in different solvents are all reduced to the same master curve.

Phillies and Clomenil [34] measured the diffusion of 67 nm polystyrene spheres through aqueous solutions followed of 139 kDa hydroxypropylcellulose at temperatures of 10 and 41 C, these being good and near-theta solvent conditions. At both temperatures, the diffusion coefficient of the spheres followed a stretched exponential $\exp(-\alpha c^{\nu })$, with ν=3/4 under good solvent conditions and ν=1 under theta conditions. Recalling the hydrodynamic scaling prediction ν=1−2x, where *x* is the concentration exponent for chain contraction, with x>0 under good solvent conditions and x≈0 under theta conditions, one sees that the observed values of ν were consistent with the hydrodynamic scaling predictions for the effect of solvent quality on $D_{p}\left(c\right)$.

### 10.6. Transition to the Melt

In its present form, the hydrodynamic scaling model refers to dilute and concentrated solutions, not to melts or plasticized melts. As the polymer concentration is increased from the dilute solution, less and less solvent is present. At some concentration, one can no longer invoke the image of a polymer solution as lines of polymer beads separated from each other by a continuum fluid. With increasing concentration, the average gap between polymer chains eventually becomes smaller than the size of a polymer molecule, rendering continuum hydrodynamic descriptions inapplicable. When continuum descriptions become inapplicable, the system switches over from solution behavior (molecules floating in a solvent) to plasticized melt behavior (solvent molecules in pockets intercalated within a mesh of polymer coils). The hydrodynamic scaling model thus predicts that there should be a qualitative change in polymer dynamic properties at some very high concentration.

Such a transition has actually been observed. Studies showing this effect include work by von Meerwall et al. [114], Pickup and Blum [115], and Kosfeld and Zumkley [116]. The diffusion coefficient of solvent molecules in polymer solutions typically follows a simple exponential dependence on concentration, for polymer concentrations up to ≈400 g/L. At larger polymer concentrations, the solvent diffusion coefficient decreases considerably more rapidly, namely as a stretched exponential in concentration with exponents in the range of 2.4–3.8. For the actual fits showing this transition, see Phenomenology of Polymer Solution Dynamics, Section 5.3.

A similar transition has been seen in segmental reorientation times [117,118] τ in some systems but not others [119]. For concentrations out to 0.3 g/g polystyrene in toluene, Viovy and Monnerie [117] and Tardiveau [118] found that τ increased gradually, perhaps by two-fold, with increasing *c*; between 0.3 and 0.5 g/g, τ increased far more rapidly, namely nearly 30-fold. On the other hand, Johnson et al. [119] studied the rotational diffusion by center-labeled polyisoprene and a small-molecule probe in polyisoprene: tetrahydrofuran for polymer matrix volume fractions ranging from 0.0 to 1.0. The matrix polymer was much more effective at delaying the rotation of the small-molecule probe than at the retarding motion of the labeled polymer. However, as τ was consistently a stretched exponential in matrix concentration, there was no indication of a transition at some elevated concentration.

A transition is also found near volume fraction 0.4 in the viscosity of hard sphere systems. An analysis of the literature supporting this observation is found in Phillies [46]. The viscosity shows a stretched-exponential increase at lower concentrations and a much sharper power-law increase at larger concentrations. The dynamic crossover is found at 0.4≤ϕ≤0.45 and $5\leq \eta_{r}\leq 15$, so the viscosity crossover is very clearly not the same as the hard sphere melting transition found at ϕ≅0.5 and $\eta_{r}≅50\pm 5$.

## 11. Summary and Directions for Future Development

This review has presented a comprehensive treatment of the hydrodynamic scaling model of polymer solution dynamics. The hydrodynamic scaling model differs from some other treatments of non-dilute polymer solutions in that it takes polymer dynamics up to high concentrations to be dominated by solvent-mediated hydrodynamic interactions. Many other models take the opposite position, namely that chain crossing constraints dominate the dynamics of non-dilute polymer solutions. We began by examining single-chain behavior, emphasizing the Kirkwood–Riseman model that forms the basis of our calculations. We then developed an extended Kirkwood–Riseman model that gives interchain hydrodynamic interactions. The model was used to generate pseudovirial series for the concentration dependencies of the self-diffusion coefficient and the low-shear viscosity. Extrapolations to a large polymer concentration were made, either based on self-similarity or on the Altenberger–Dahler positive-function renormalization group. The observed stretched-exponential concentration dependencies were predicted. An inferred fixed-point structure for the renormalization group led to a two-parameter temporal scaling ansatz from which the frequency dependencies of the storage and loss moduli were inferred.

This section notes possible directions for future research. Topics needing further consideration include: (i) inclusion of intrachain hydrodynamic interactions; (ii) incorporation of segmental dynamics; (iii) explanation of the solution-like–melt-like viscosity transition and (perhaps a different reflection of the same phenomenon) the neutral polymer slow mode, (iv) extension, refinement, or replacement of the Positive-Function Renormalization Group approach to extrapolating to elevated concentrations; (v) direct treatment of frequency or time dependence, leading to results for the plateau modulus, steady state compliance, characteristic shear rate, and the Cox–Merz rule. On a longer time scale, note that (vi) the treatment of the polymer as its own (viscoelastic) solvent leads to the modeling of melt systems; and (vii) the extension to polyelectrolyte systems.

The hydrodynamic calculations considered above only treat interactions between polymer chains. Intrachain hydrodynamic interactions have not yet been incorporated into the model, though Kirkwood and Riseman [5] show how this incorporation might advance. These interactions are important because the motion of each bead partially entrains the surrounding solvent, so that the beads near the center of a polymer coil are less effective than might have been expected upon creating fluid motion in the surrounding solvent.

The model as treated above remains true to the Kirkwood–Riseman spirit, namely the fact that it focuses on whole-body translations and rotations, but neglects internal chain motions. Experimental techniques including dielectric relaxation and NMR have been used to examine segmental dynamics, the relative motions of parts of a longer polymer chain, but the theoretical interpretation of these measurements in terms of the hydrodynamic scaling model is lacking.

The experimental development that led to the hydrodynamic scaling model began with probe diffusion, the measurements of the motion of rigid spheres or other particles through polymer solutions. The current theoretical treatment is appropriate when the probe and polymer coils are similar in size. If the probe is much smaller than the matrix polymers, probe diffusion may be enhanced by the relative motion of parts of nearby chains, i.e., by segmental dynamics. The extension of the hydrodynamic scaling model to treat segmental dynamics would advance our understanding of both of these issues.

Some concentration dependence issues are not yet resolved. Phillies and Quinlan demonstrated for hydroxypropylcellulose the existence of a solution-like–melt-like transition, in which the concentration dependence of the viscosity switches over from a stretched-exponential to a power law in concentration. Similar transitions can be observed for η(c) of many but not all polymers. However, the transition is not universal, in the sense that it occurs in different polymers at very different concentrations, regardless of whether the transition concentration is expressed as a polymer weight concentration (approximately a volume fraction) or as a concentration in natural units c[η], [η] being the polymer’s intrinsic viscosity. Furthermore, the transition is observed for solutions of hard and soft spheres in small-molecule solvents. The experiment thus rules out interpretations of the transition as arising from long-chain topological effects. Quasi-elastic light scattering from other polymer and probe solutions finds the appearance of a slow mode in the light scattering spectrum at elevated concentrations, without an accompanying significant change in the light scattering static intensity [120], ruling out cluster formation as an explanation for the slow mode. The slow mode appears in the light-scattering spectrum at approximately the concentration at which the solution-like–melt-like transition is observed in the viscosity, suggesting that the two effects have a common origin, perhaps arising from the fixed-point structure of the positive-function renormalization group for this problem.

The extension of the hydrodynamic calculations to elevated concentrations was based on the positive-function renormalization group. The renormalization group calculation does yield answers that agree with the experiment, but the path is obscured by the indirect nature of renormalization group methods. A clearer calculation might also permit one to calculate higher-order corrections to the basic renormalization group forms.

The model as presented herein gives an average diffusion coefficient or a low-frequency viscosity. A two-variable renormalization group ansatz was introduced to predict the functional form of the frequency dependencies of the storage and loss moduli and the dependence of shear thinning on the shear rate. The prediction is an ansatz, not a theoretical derivation. The replacement of the ansatz with a full calculation should replace qualitative statements with quantitative predictions. In particular, the predicted functional forms for the frequency dependencies embody a series of parameters, each with concentration and molecular weight dependencies. A direct calculation should give numerical values for these parameters. Also, polymer solution dynamics includes linear and non-linear viscoelastic effects. The analytic calculation would predict the plateau modulus, steady-state compliance, and characteristic shear rate for shear thinning. One might also reasonably expect that such calculations would both explain the approximate validity of the Cox–Merz rule and predict the quantitative corrections to that rule.

Finally, while the simulational tests of concentrated solutions are non-trivial, because vast numbers of solvent molecular must be included, they are not impossible. The simulational studies of whole-chain translation and rotation in melts, including correlations in the whole-body translations and rotations of neighboring chains, would speak to the validity of the models.

Calculations on melts and polyelectrolyte systems remain well into the future.

## Data Availability

All data are in the main body of the paper.
